# Single-nucleus and bulk RNA sequencing reveal cellular and transcriptional mechanisms underlying lipid dynamics in high marbled pork

**DOI:** 10.1038/s41538-023-00203-4

**Published:** 2023-06-02

**Authors:** Liyi Wang, Xueyan Zhao, Shiqi Liu, Wenjing You, Yuqin Huang, Yanbing Zhou, Wentao Chen, Shu Zhang, Jiying Wang, Qiankun Zheng, Yizhen Wang, Tizhong Shan

**Affiliations:** 1 College of Animal Sciences, Zhejiang University, Hangzhou, China; 2 Key Laboratory of Molecular Animal Nutrition (Zhejiang University), Ministry of Education, Hangzhou, China; 3 Key Laboratory of Animal Feed and Nutrition of Zhejiang Province, Hangzhou, China; 4 Institute of Animal Science and Veterinary Medicine, Shandong Academy of Agricultural Sciences, Jinan, 250100 China; 5 DELISI GROUP Co. Ltd, Weifang, Shandong China

**Keywords:** Transcriptomics, Animal physiology, Biomaterials - cells

## Abstract

Pork is the most consumed meat in the world, and its quality is associated with human health. Intramuscular fat (IMF) deposition (also called marbling) is a key factor positively correlated with various quality traits and lipo-nutritional values of meat. However, the cell dynamics and transcriptional programs underlying lipid deposition in highly marbled meat are still unclear. Here, we used Laiwu pigs with high (HLW) or low (LLW) IMF contents to explore the cellular and transcriptional mechanisms underlying lipid deposition in highly-marbled pork by single-nucleus RNA sequencing (snRNA-seq) and bulk RNA sequencing. The HLW group had higher IMF contents but less drip loss than the LLW group. Lipidomics results revelled the changes of overall lipid classes composition (e.g., glycerolipids including triglycerides, diglycerides, and monoglycerides; sphingolipids including ceramides and monohexose ceramide significantly increased) between HLW and LLW groups. SnRNA-seq revealed nine distinct cell clusters, and the HLW group had a higher percentage of adipocytes (1.40% *vs*. 0.17%) than the LLW group. We identified 3 subpopulations of adipocytes, including PDE4D^+^/PDE7B^+^ (in HLW and LLW), DGAT2^+^/SCD^+^ (mostly in HLW) and FABP5^+^/SIAH1^+^ cells (mostly in HLW). Moreover, we showed that fibro/adipogenic progenitors could differentiate into IMF cells and contribute to 43.35% of adipocytes in mice. In addition, RNA-seq revealed different genes involved in lipid metabolism and fatty acid elongation. Our study provides new insights into the cellular and molecular signatures of marbling formation; such knowledge may facilitate the development of new strategies to increase IMF deposition and the lipo-nutritional quality of high marbled pork.

## Introduction

Meat is an important source of protein for humans, and it directly impacts human health and is closely associated with a better life for humans. People are pursuing ‘less but better’ meat to tackle the sustainability challenges of meat production and consumption^1,2^. Many quality aspects can be used to define ‘better meat’, such as eating quality, including flavour, tenderness, and juiciness^3^. Marbling (also called intramuscular fat (IMF) deposition or accumulation) is important for estimating the potential eating quality of pork loins, and it gives meat its particular flavour, succulence and tenderness^4,5^. In humans, fat infiltration in skeletal muscle is often accompanied by ageing and metabolic or nonmetabolic diseases, such as obesity, diabetes, and myosteatosis^6–8^. However, the cell sources and regulatory mechanism of IMF deposition are still unknown. Hence, it is necessary to explore the regulatory mechanism of IMF deposition and its impact on lipid metabolism in skeletal muscle.

Pork is the most produced and consumed meat worldwide. In particular, China is the world’s largest pork market and survey results show that nearly 60% of Chinese consumers eat pork at least three to five times a week. However, obese and lean-type pig breeds show obvious differences in lipid deposition, and the IMF content of lean-type pig breeds is always low. Hence, how to appropriately increase IMF content while ensuring lean meat percentage and without increasing subcutaneous fat content to produce high-quality pork is a key challenge in pig production. With the continuous development of high-throughput sequencing, an increasing number of technologies have been reported to identify the cell sources and transcriptional heterogeneity in skeletal muscle, including single-cell RNA-seq (scRNA-seq)^9^ and spatial transcriptomics (ST)^10^. They have been used to identified several cell types that contribute to the formation of IMF, including satellite cells (SCs), fibro/adipogenic progenitors (FAPs), mesenchymal stem cells (MSCs), fibroblasts, endothelial cells (ECs), pericytes, side population cells (SPs), PW1^+^/Pax7^−^ interstitial cells (PICs), and myeloid-derived cells. Due to the size of myotubes and myofibers, it is difficult to detect larger cell types in skeletal muscle. Interestingly, different to scRNA-seq and ST, single-nucleus RNA-seq (snRNA-seq)^11^ is another high-resolution transcriptome method that can assay both mononuclear and multinucleated cells together. However, recent studies using by snRNA-seq have mostly focused on mice^12,13^, and there is still no study on livestock and poultry using snRNA-seq.

Local Chinese pig species always have a higher IMF content than lean breeds of pigs, and they can serve as good models for studying the IMF deposition mechanism. When the IMF content is larger than 8%, the pork is always considered snowflake pork (high marbled pork). Laiwu pig is an excellent local Chinese pig species; it has a high IMF content, and the average IMF content is 11.6%. However, the IMF content of Laiwu pigs varies greatly among individuals, and the differential mechanism is still unclear. Hence, it can serve as an appropriate animal model for studying the cell heterogeneity and regulatory mechanism of IMF deposition. In this study, we used Laiwu pigs with high and low IMF content to serve as a study model, and we explored the lipid dynamics, myonuclear heterogeneity, transcriptomic profiles, and differential mechanism of IMF in skeletal muscle through multiomics (lipidome, snRNA-seq, and RNA-seq). Our findings will provide new insights into the cytological and molecular mechanisms of marbling formation, which may provide a foundation for the production of high-quality snowflake pork.

## Results

### The importance of marbling in meat quality

**Fig. 1:**
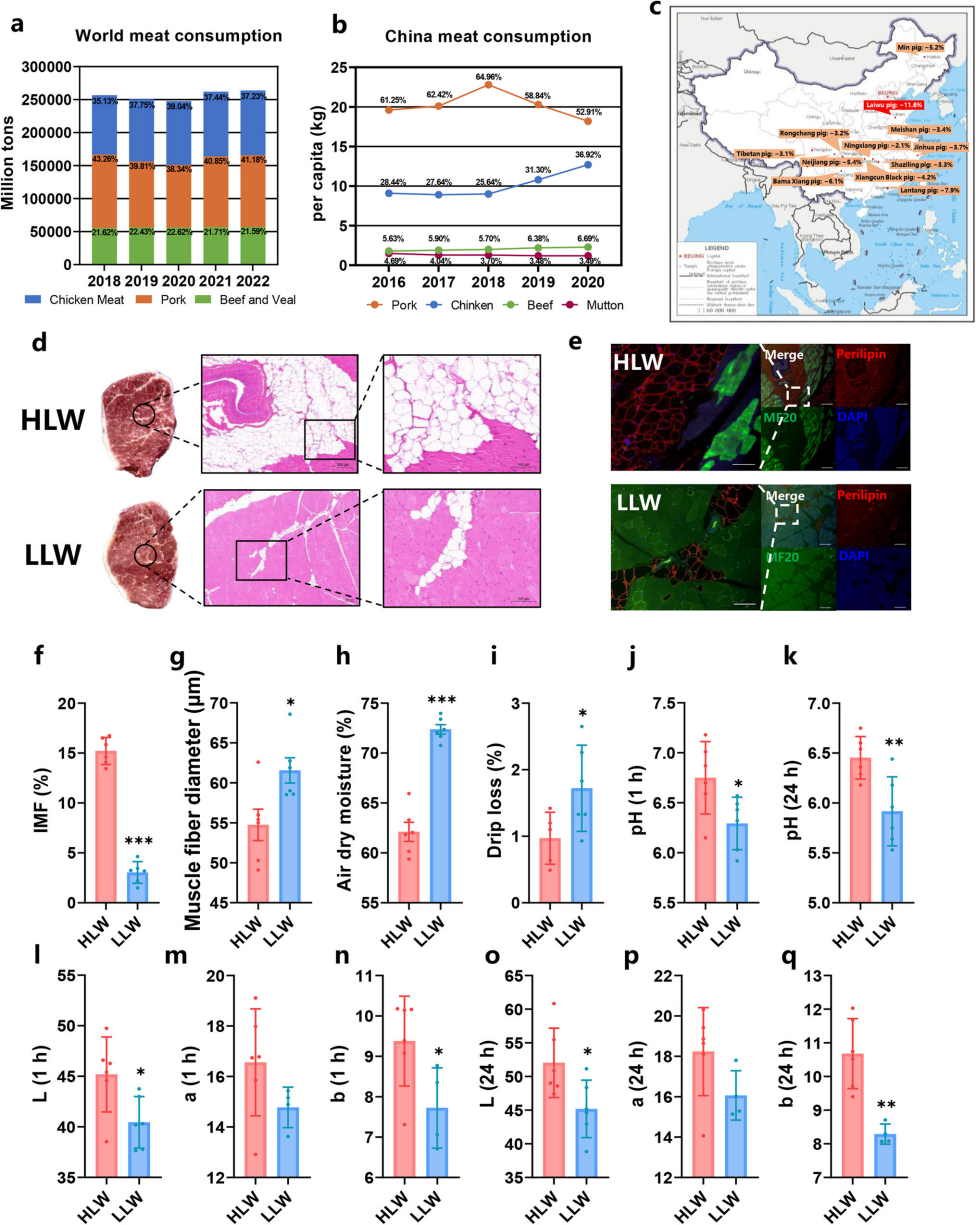
IMF content and meat quality between the HLW and LLW groups. **a** World meat consumption patterns. Sources from United States Department of Agriculture-Foreign Agricultural Service. **b** China meat consumption patterns. Sources from National Bureau of Statistics of China-China Statistical Yearbook (2021). **c** IMF levels in local Chinese pig breeds. **d** Representative LDM tissues and H&E staining of LDM sections from the HLW and LLW groups. Scale bars, 200 and 100 μm, respectively. **e** LDM tissues stained with the adipogenic marker perilipin (red), muscle fiber marker MF20 (green) and DAPI (blue) in different groups. **f**–**q** Meat quality (IMF content, muscle fiber diameter, air dry moisture, drip loss, pH, L, a, and b value at 1 h and 24 h) in the HLW and LLW groups (*n* = 6). Error bars represent SEM. ^*^P < 0.05, ^**^P < 0.01, two-tailed Student’s *t*-test.

Beef, pork and chicken constitute nearly 90% of global meat production and in the past five years, pork is still the number one meat consumed in the world (average 40.67%) despite the impact of African swine fever (Fig. 1a). In China, pork consumption plays a major role in meat consumption (average 60.08%) (Fig. 1b). Local Chinese pig breeds always have a high IMF content, and Laiwu pigs have the highest IMF content (almost 11.6%) (Fig. 1c). IMF content is an important meat quality value for pork quality and it positively affects sensory properties, including tenderness, juiciness, flavour, and oiliness (Supplementary Fig. 1a). Hence, we chose Laiwu pigs as our study model to explore IMF deposition in pigs.

### Different IMF deposition and meat quality in Laiwu pigs

The results showed that there were no changes in carcass weight and backfat thickness between the two groups (Supplementary Fig. 1b, c). Compared with the LLW group, the HLW group had significant marbling and higher IMF content in longissimus dorsi muscle (LDM) (Fig. 1d, f). The immunofluorescence results also showed more lipid-containing cells in the LDM of the HLW group (Fig. 1e). Meanwhile, the muscle fiber diameter, air dry moisture, and drip loss were reduced in the HLW group (Fig. 1g–i). In addition, the pH value is an important indicator to reflect the muscle contraction and glycolysis rate of pigs after slaughter and meat color is one of the most important factors that control the sensory quality of pork^14^. We found the HLW group had a higher pH (1 h and 24 h after slaughter), L (1 h and 24 h after slaughter), and b (1 h and 24 h after slaughter) value (meat color value, L value refers to lightness and b value refers to yellowness) than the LLW group (Fig. 1j–q). However, there was no significant difference in a (1 h and 24 h after slaughter) value (meat color value, a value refers to redness) and biochemical indices in the serum (Fig. 1m, p, and Supplementary Fig. 1d–o). These data demonstrated that the HLW group had a higher IMF content and different meat quality compared with the LLW group.

### Changes in the overall lipid profiles in the LDM of HLW pigs

**Fig. 2:**
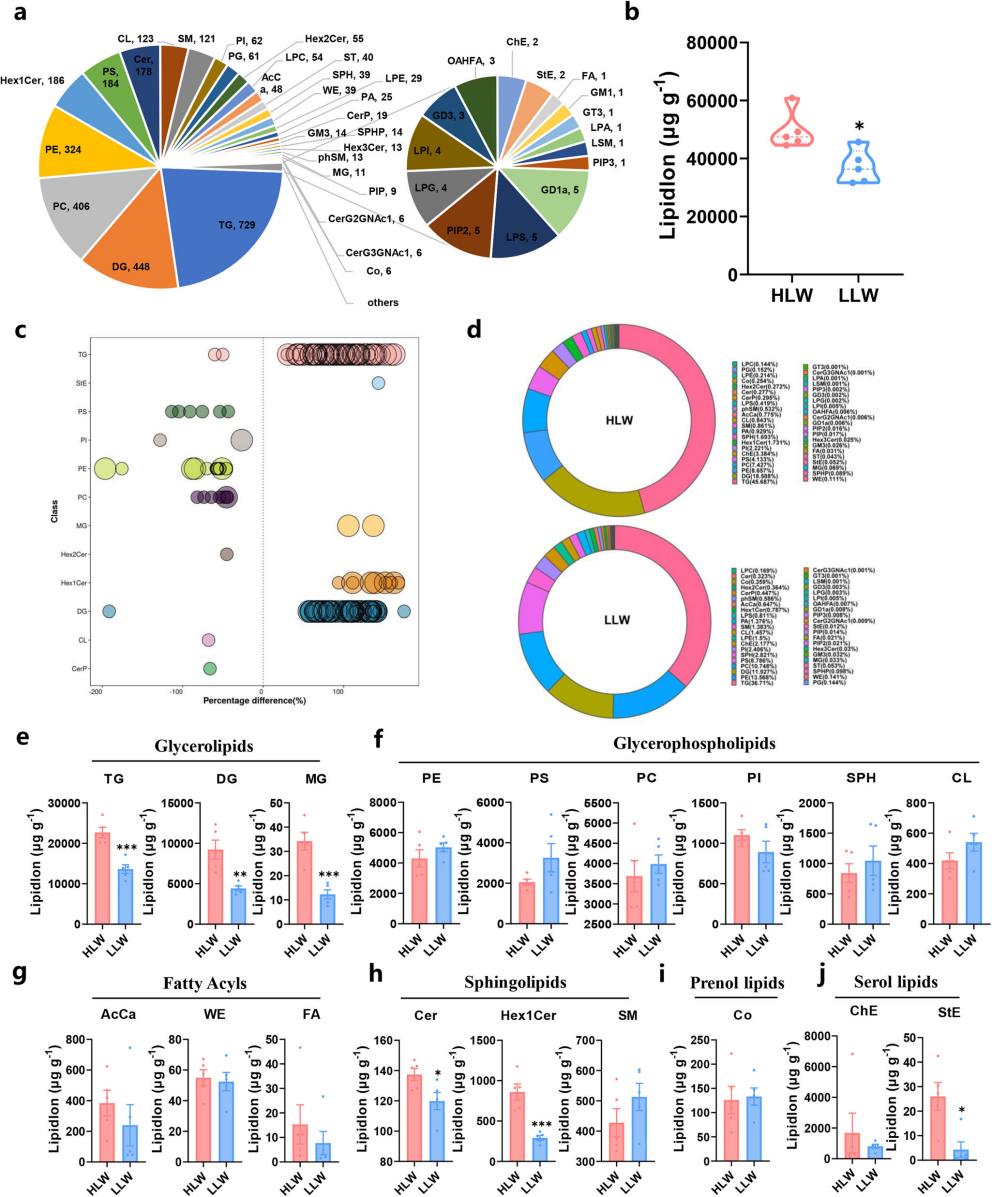
Changes in the overall lipid composition and distribution in LDM in different groups. **a** Distribution of lipid classes that were considered for subsequent analysis in all of the samples detected by LC-MS/MS. **b** The content of total lipids (the amount of all quantified lipid molecules in the same sample is added) in LDM of Laiwu pigs. **c** The percentage difference in lipid class between different groups. Each dot represents a lipid species, and the dot size indicates significance in which bigger dot refers to *P* < 0.01 and a smaller dot refers to 0.01 < *P* < 0.05 (*n* = 5). **d** Proportion of different lipid species between the HLW and LLW groups. The contents of glycerolipids (**e**), glycerophospholipids (**f**), fatty acyls (**g**), sphingolipids (**h**), prenol lipids (**i**), and serol lipids (**j**) in LDM from the HLW and LLW groups (*n* = 5). Error bars represent SEM. ^*^P < 0.05, ^**^P < 0.01, two-tailed Student’s *t*-test. L, lightness; a, redness; b, yellowness.

To further determine the changes in the overall lipid composition and distribution in snowflake pork, we isolated LDM and applied mass spectrometry-based lipidomic analysis. In total, we detected over 3301 different lipid species in LDM, consisting of 729 triglycerides (TGs), 448 diglycerides (DGs), 406 phosphatidylcholines (PCs), 324 phosphatidylethanolamines (PEs), and other lipid classes (Fig. 2a). The orthogonal partial least squares discrimination analysis (OPLS-DA) plot showed a clear separation of the HLW and LLW groups (Supplementary Fig. 2a). In the HLW group, the levels of total lipids were higher than those in the LLW group (Fig. 2b). Overall, the concentrations of glycerolipids and sphingolipids were higher in the HLW group (Supplementary Fig. 2b). The proportion of TGs in the HLW group (45.69%) was higher than that in the LLW group (36.71%), but the proportion of PEs in the HLW group (8.66%) was lower than that in the LLW group (13.57%) (Fig. 2c, d). As shown in Fig. 2e, h, j, at the lipid class level, TGs (*P* < 0.001); DGs (*P* < 0.01); monoglycerides (MGs, *P* < 0.001), a type of glycerolipid; ceramides (Cers, *P* < 0.05), monohexose ceramide (Hex1Cers, *P* < 0.001), a type of sphingolipid; and stigmasterol esters (StEs, *P* < 0.05), a type of serol lipid, were increased in the HLW group. In addition, there was no significant difference in other lipid classes (Fig. 2f–j and Supplementary Fig. 2c–e). At the lipid species level, the heatmap showed that differential lipid species were mostly in TGs, DGs, and PEs between the HLW and LLW groups (Supplementary Fig. 2f).

We next explored the acyl chain content in TGs, which indicated the fatty acid composition in lipids. As shown in Supplementary Fig. 3a, saturated fatty acids (SFAs) associated with TG acyl chains, including C6:0, C8:0, C10:0, C12:0, C14:0, C16:0, C20:0, C22:0, and C30:0 were significantly increased in the HLW group. Monounsaturated fatty acids (MUFAs), including C10:1, C12:1, C16:1, C18:1, and C20:1 were largely increased in the HLW group. The concentrations of polyunsaturated fatty acids (PUFAs), including C10:2, C10:4, C12:2, C12:3, C14:3, C14:4, C16:2, C18:2, C18:3, C18:4, C20:2, C20:4, C22:5, C22:6, C24:2, C34:4, C36:4, C36:7, C38:2, C38:5, and C58:13, increased significantly in the HLW group. In addition, large increases in the concentrations of odd-numbered fatty acyl chains, including C9:0, C11:0, C19:0, C23:0, C11:1, C17:1, C19:1, C23:1, C35:4, C37:8, C41:7, C47:9, C49:12, C55:8, C57:11, and C59:11, were observed in the HLW group (Supplementary Fig. 3b). Moreover, we found that the contents of SFAs, MUFAs, and PUFAs were also significantly increased in the TG acyl chain (Supplementary Fig. 3c) and the percentages of SFAs and MUFAs were also significantly increased in the HLW group (Supplementary Fig. 3d). The proportion of TGs with a higher number of carbon atoms (>48) and double bonds (<10) increased significantly in the HLW group (Supplementary Fig. 3e, f). These results suggested significant differences in the fatty acyl chain composition associated with TGs between the different groups.

### snRNA-seq identified distinct cell populations in LDM between HLW and LLW pigs

**Fig. 3:**
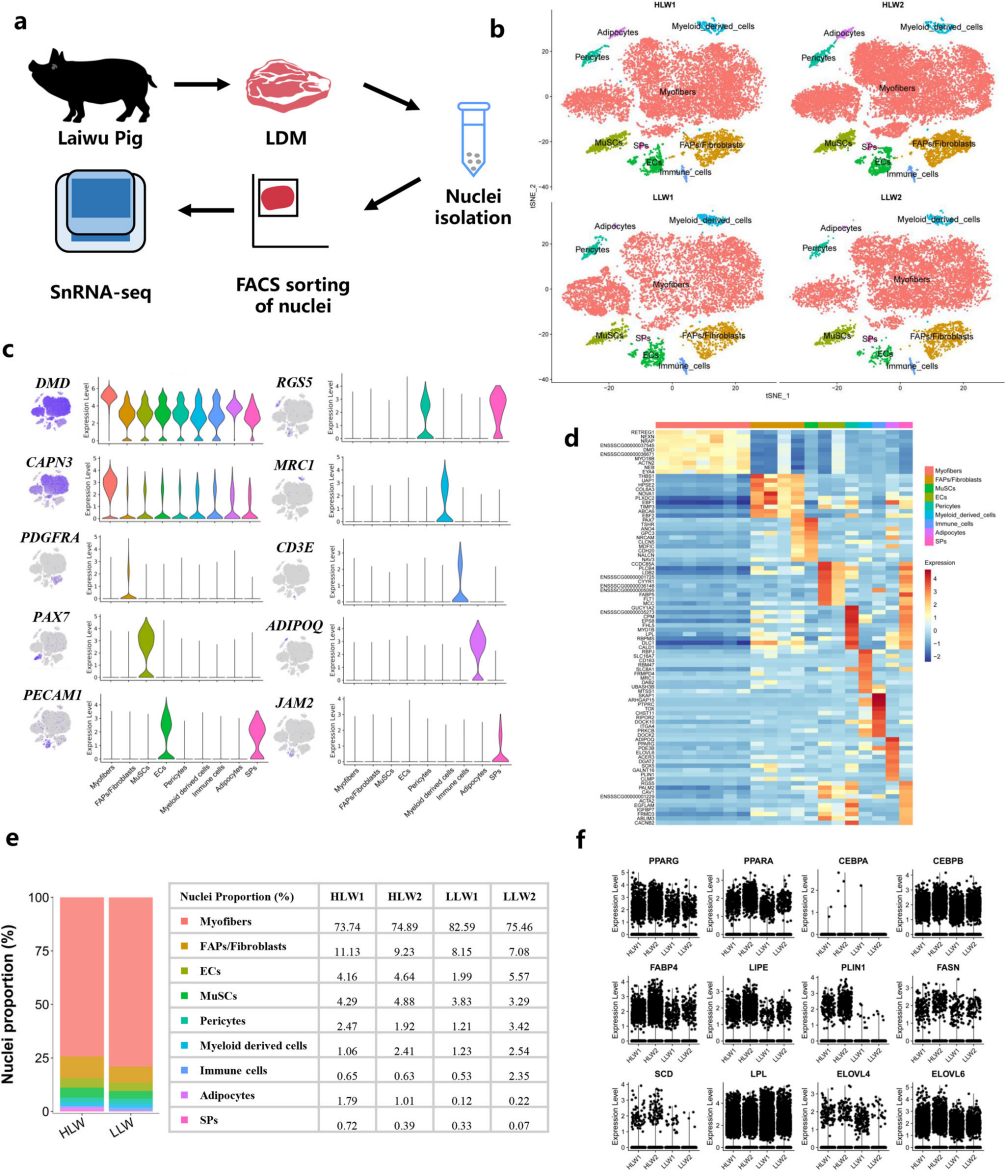
snRNA-seq identifies distinct cell populations in the LDM of the HLW and LLW groups. **a** Scheme of the experimental design for snRNA-seq on LDM nuclei. **b** tSNE visualization of all of the isolated single nuclei from HLW and LLW muscle coloured by cluster identity. **c** tSNE and violin plot displaying the expression of selected marker genes for each cluster of nuclei. **d** Heatmap representing the top 10 most differentially expressed genes between cell clusters identified. Each lane represents a subcluster. **e** Nuclear proportion in each cluster in HLW and LLW muscles. Each cluster is colour-coded. **f** Violin plot showing the expression of adipogenic master genes, mature adipocyte marker genes, and lipid metabolism-related genes.

To explore the cell dynamics of snowflake pork, we developed a new snRNA-seq method for nuclei extraction from LDM tissue using the 10× Genomics Chromium platform (Fig. 3a). The results obtained from Cell Ranger analyses showed that the estimated number of cells in this study was 56108, the fraction of reads in cells was 77.9%, mean reads per cell was 14740, median genes per cell was 954, and median UMI counts per cell was 1611 (Supplementary Fig. 4a). Following the quality control of snRNA-seq data, we retained 47273 nuclei from 4 individual libraries, comprising 28418 nuclei from 2 HLW and 18855 nuclei from 2 LLW for the downstream analysis (Supplementary Fig. 4b). Using the Seurat package (3.1.1), aggregated and normalized snRNA-seq data were then subjected to clustering to identify the cell types, which were shown in t-distributed stochastic neighbour embedding (t-SNE) plots (Fig. 3b). Based on the expression of lineage specific markers, we identified 9 different clusters of nuclei, including myofibers (*DMD* and *CAPN3*), FAPs/fibroblasts (*PDGFRA*), muscle satellite cells (MuSCs) (*PAX7*), ECs (*PECAM1*), pericytes (*RGS5*), myeloid derived cells (*MRC1*), immune cells (*CD3E*), adipocytes (*ADIPOQ*), and SPs (*JAM2*) (Fig. 3c). The tSNE and violin plot also displayed the expression of the myosin heavy chain genes (*MYH7*, *MYH2*, *MYH1*, and *MYH4*) (Supplementary Fig. 4c). The heatmap showed the top 10 most variably expressed genes between the 9 cell clusters (Fig. 3d).

Next, we analysed the difference in cell populations in the LDM of the HLW and LLM groups. Compared with the LLW group, the HLW group had a lower percentage of myofiber nuclei (74.32% *vs*. 79.02%) and a higher percentage of MuSCs (4.59% *vs*. 3.56%). Importantly, the HLW group had a higher percentage of adipocytes (1.40% *vs*. 0.17%) than the LLW group. In addition, for other nonmyofiber nuclei, such as FAPs/fibroblasts, ECs, and SPs we also found increases in these cell types in the HLW group (FAPs/fibroblasts: 11.13% and 9.23%, ECs: 4.16% and 4.64%, and SPs: 0.72% and 0.39%) (Fig. 3e).

To determine the difference in IMF deposition of the two groups, we examined the expression of adipogenesis- and lipid metabolism-related genes. As shown in Fig. 3f, the expression of adipogenic master genes (*PPARG*, *PPARA*, *CEBPA*, and *CEBPB*), mature adipocyte marker genes (*FABP4*, *LIPE*, and *PLIN1*), and lipid metabolism-related genes (*FASN*, *SCD*, *ELOVL4*, and *ELOVL6*) was increased, but the expression of *LPL* was decreased in the HLW group. Additionally, we also observed that the expression of fatty acid metabolism-related genes such as *ACAA1*, *FABP3*, *FABP4*, *FABP5*, *CPT1A*, *ACSL4*, and *ELOVL1* was upregulated but *HACD1* was downregulated in the HLW group (Supplementary Fig. 4d). Moreover, we also found that these genes were expressed in SP, adipocyte, and EC cell types (Supplementary Fig. 4e). There was a difference in the expression of sphingolipid metabolism-related genes (*SGPP1*, *KDSR*, *GALC*, *ARSA*, and *GAL3ST1*) between the two groups and different cell clusters (Supplementary Fig. 4f, g). These results suggested that the cell populations had significant differences in the LDM of different Laiwu pigs, and IMF deposition and alterations in lipids might be due to changes in cell clusters and the expression of related genes.

To identify the cause of the decreases in muscle fiber diameter, we performed a subcluster analysis and explored the heterogeneity of myofiber nuclei. t-SNE plots showed the distribution in different subclusters of myofiber nuclei (Supplementary Fig. 5a). By comparing gene expression in myofiber nuclei, we characterized 6 subclusters in myofiber nuclei, including type I myonuclei (*MYH7*), type IIa myonuclei (*MYH2*), type IIx myonuclei (*MYH1*), type IIb myonuclei (*MYH4*), neuromuscular junction (NMJ, *ABLIM2*)^15^ and myotendinous junctions (MTJ, *ANKRD1*)^16^ (Supplementary Fig. 5b). In the proportion of these subclusters, we observed that the percentage of type IIa myonuclei had an increased tendency (29.37% *vs*. 23.95%) while the percentage of type IIb myonuclei had a decreased tendency (38.56% *vs*. 43.75%) in the HLW group (Supplementary Fig. 5c). The heatmap showed the top 10 most variably expressed genes between the 6 cell subclusters (Supplementary Fig. 5d). We found that oxidation-related genes (*COX5A*, *COX5B*, *COX8A*, and *CPT1A*) had relatively higher expression levels, but glycolysis-related genes (*PKM*, *PFKM*, and *HK2*) had relatively lower expression levels in the HLW group (Supplementary Fig. 5e). In addition, myofiber type transformation-related genes (*STK11*, *PPARGC1A*, *SIRT1, PPARD, ADIPOQ, MAPK1, NFATC1*, and *HDAC1*) were also relatively different between the two groups (Supplementary Fig. 5f). These data showed the significantly heterogeneity in myofiber nuclei in two groups and the decrease in muscle fiber diameter in the HLW group might result from the changes of muscle fiber type and altered expression of muscle fiber type related genes.

### Clustering analysis identified subpopulations and transcriptional dynamics of adipocytes

**Fig. 4:**
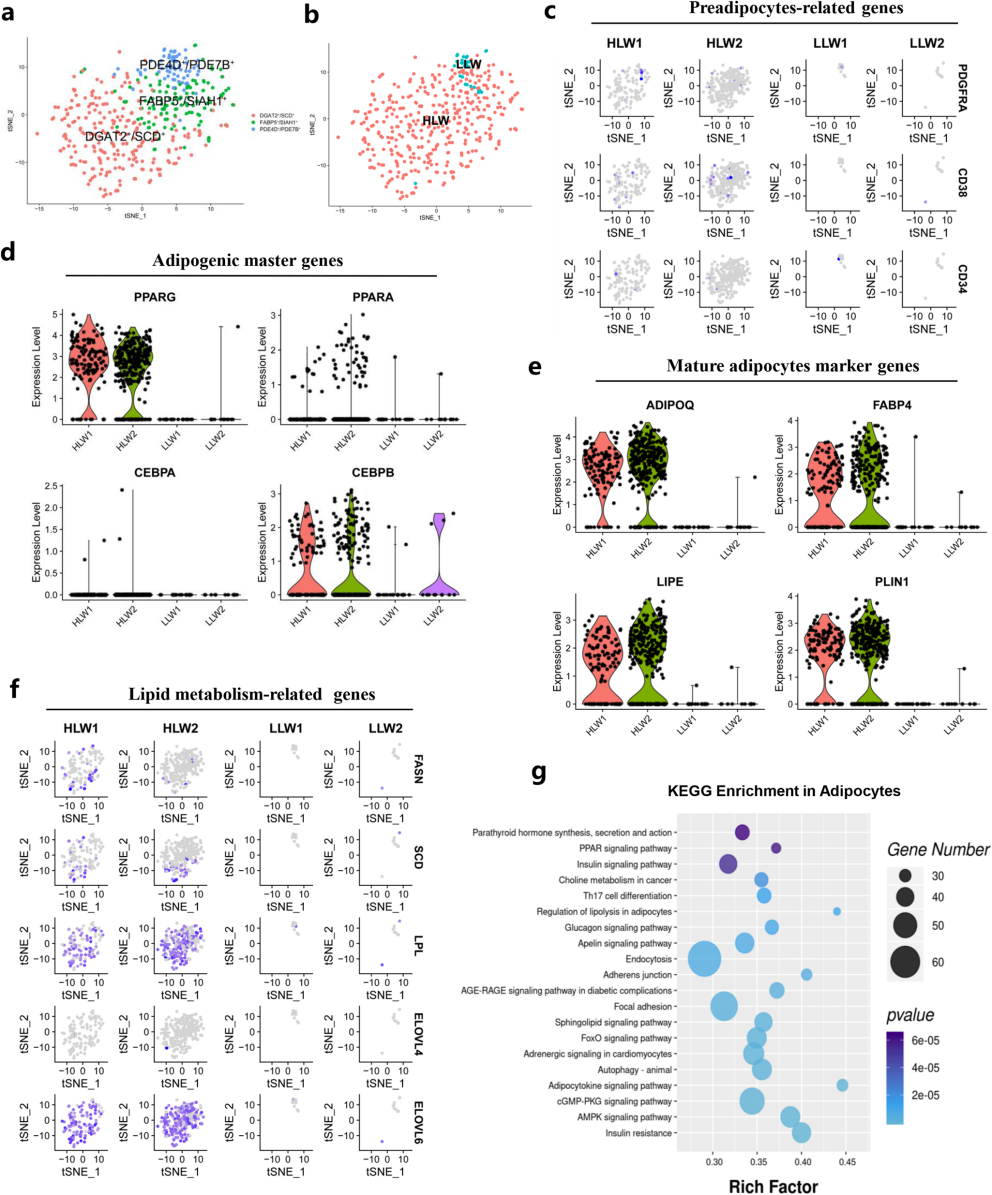
Clustering and transcriptional dynamics of adipocytes. **a** tSNE plot displaying the isolated single nuclei in three subclusters. **b** tSNE plot showing the isolated single nuclei from HLW and LLW muscle. **c** tSNE plot showing the expression of preadipocyte-related genes in different groups. **d** Violin plot showing the expression of adipogenic master genes in different groups. **e** Violin plot displaying the expression of mature adipocyte marker genes in different groups. **f** Violin plot displaying the expression of lipid metabolism-related genes in different groups. **g** KEGG enrichment analysis of genes in adipocytes. Statistical analysis was performed using two-tailed Student’s *t*-test.

To investigate the cellular mechanism for high-marbled pork, we performed a subcluster analysis on adipocytes. We identified 3 subclusters including DGAT2^+^/SCD^+^ subclusters, FABP5^+^/SIAH1^+^ subclusters, and PDE4D^+^/PDE7B^+^ subclusters based on their most variably expressed genes (Fig. 4a and Supplementary Fig. 6a). Interestingly, we found that compared with the LLW group, the HLW group had a largely higher percentage of DGAT2^+^/SCD^+^ subclusters and FABP5^+^/SIAH1^+^ subclusters (Fig. 4b), which meant that the difference in IMF deposition mainly depends on DGAT2^+^/SCD^+^ subclusters and FABP5^+^/SIAH1^+^ subclusters. The expression levels of preadipocyte-related genes (*PDGFRA*, *CD38*, and *CD34*), adipogenic master genes (*PPARG*, *PPARA*, *CEBPA*, and *CEBPB*), mature adipocyte marker genes (*ADIPOQ*, *FABP4*, *LIPE*, and *PLIN1*), and lipid metabolism-related genes (*FASN*, *SCD*, *LPL*, *ELOVL4*, and *ELOVL6*) were all increased in the HLW group (Fig. 4c–f). In addition, we also observed that the expression of fatty acid metabolism-related genes (*ACAA1*, *FABP3*, *FABP4*, *FABP5*, *ACSL4*, *HACD1*, and *ELOVL1*) and sphingolipid metabolism-related genes (*SGPP1*, *KDSR*, *GALC*, *ARSA*, and *GAL3ST1*) was upregulated in the HLW group (Supplementary Fig. 6b, c).

Functional enrichment analyses using KEGG pathways revealed a significant enrichment of the PPAR signalling pathway, insulin signalling pathway, and AMPK signalling pathway in adipocytes (Fig. 4g); the calcium signalling pathway, insulin resistance, PPAR signalling pathway, and AMPK signalling pathway in DGAT2^+^/SCD^+^ subclusters (Supplementary Fig. 6d); the TGF-beta signalling pathway, thermogenesis, cGMP-PKG signalling pathway, and AMPK signalling pathway in FABP5^+^/SIAH1^+^ subclusters (Supplementary Fig. 6e); and the calcium signalling pathway, AMPK signalling pathway, insulin resistance, and cAMP signalling pathway in PDE4D^+^/PDE7B^+^ subclusters (Supplementary Fig. 6f). These data revealed 3 subpopulations of adipocytes, including PDE4D^+^/PDE7B^+^ (in HLW and LLW), DGAT2^+^/SCD^+^ (mostly in HLW) and FABP5^+^/SIAH1^+^ subclusters (mostly in HLW), in which different subclusters play different roles in IMF deposition and we speculated the difference in IMF deposition mainly relies on DGAT2^+^/SCD^+^ subclusters and FABP5^+^/SIAH1^+^ subclusters. means

### Clustering, pseudotime analysis, and cell–cell communication analysis revealed the cell origins of IMF

**Fig. 5:**
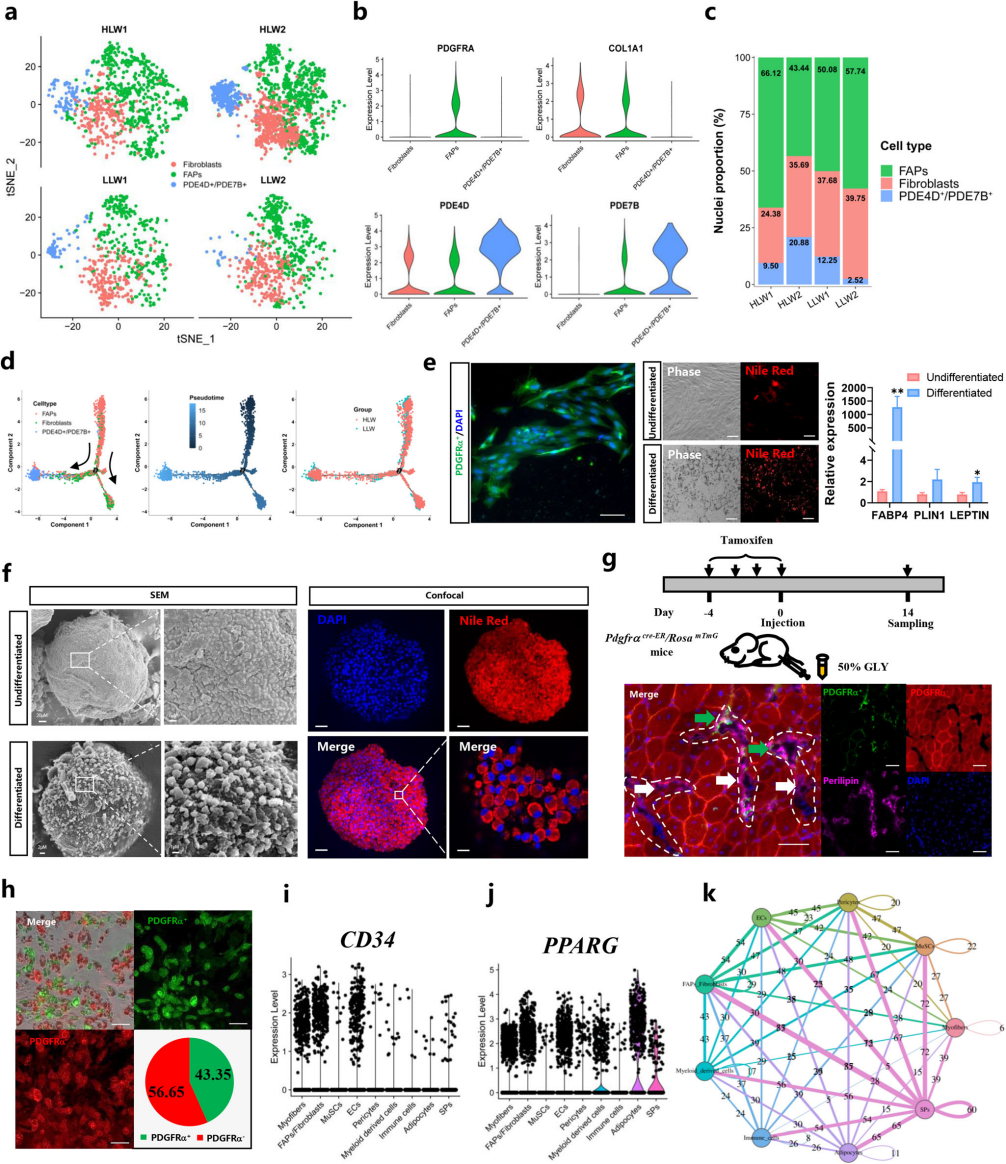
Clustering, pseudotemporal trajectories and cell–cell communication analysis revealed the networks of IMF cell types. **a** tSNE plot showing three subclusters of the isolated single nuclei from HLW and LLW muscle. **b** Violin plot displaying the expression of selected marker genes for each subcluster of nuclei. **c** Nuclear proportion in each subcluster in HLW and LLW muscles. Each cluster is colour-coded. **d** Pseudotime ordering of all of the FAP/fibroblast nuclei of subcluster FAPs, fibroblasts, and PDE4D^+^/PDE7B^+^. Each dot represents one nucleus (colour-coded by its identity), and each branch represents one cell state. Pseudotime is shown coloured in a gradient from dark to light blue, and the start of pseudotime is indicated. Activation of the FAP cluster can lead to fibroblast fate or to PDE4D^+^/PDE7B^+^ fate. **e** Fluorescence, phase and adipogenesis-related mRNA relative expression of undifferentiated and differentiated FAPs stained with Nile Red (red) and DAPI (blue). White circles: cells with lipid droplets. Scale bars, 100 μm. **f** Scanning electron microscope and Nile Red staining images showed the structure of lipid droplets in undifferentiated and differentiated FAPs by 3D culture. Scale bars, 100 μm and 400 μm, respectively. **g** Scheme of injecting GLY into the TA of *Pdgfrα*^*cre-ER*^*/ROSA*^*mTmG*^ mice for 14 days after injecting tamoxifen for 4 days and fluorescence of TA sections stained with Perlipin (pink) and DAPI (blue). White areas: lipid droplets; arrow areas: *Pdgfrα*^*-*^ lipid droplets. Scale bars, 100 μm and 200 μm, respectively. **h** Fluorescence, phase and cell proportion of differentiated IMF cells isolated from the muscles of *Pdgfrα*^*creER*^*/ROSA*^*mTmG*^ mice. Scale bars, 100 μm. **i**, **j** Violin plot displaying the expression of preadipocyte-related genes and adipogenic master genes in muscle nuclei. **k** Cell–cell communication analysis of clusters.

To investigate the cellular origin of IMF, we then analysed other nonadipocyte nuclei. Previous studies have shown that FAPs are the main source of IMF cells^17,18^. Hence, we first applied subcluster analysis on FAPs/fibroblasts. In total, we identified 3 subclusters in FAPs/fibroblasts according to the expression of specific markers, including FAPs (*PDGFRA*), fibroblasts (*COL1A1*), and PDE4D^+^/PDE7B^+^ subclusters (*PDE4D* and *PDE7B*) (Fig. 5a, b). We found that the proportion of nuclei in fibroblasts in the HLW group had a decreased tendency compared to the LLW group (Fig. 5c). At the gene expression level, there were relative differences in preadipocyte-related genes (*PDGFRA* and *CD38*), adipogenic master genes (*PPARG* and *PPARA*), mature adipocyte marker genes (*ADIPOQ*, *FABP4*, *LIPE*, and *PLIN1*), lipid metabolism-related genes (*FASN*, *ELOVL4*, *ELOVL6*), and FAP master genes (*TGFBR1*, *TGFB2, SMAD2*, and *SMAD3*) between the two groups (Supplementary Fig. 7a). In addition, the expression of marker genes for the adipocyte subclusters that we have identified was higher in the HLW group (Supplementary Fig. 7b).

Functional enrichment analyses using KEGG pathways revealed a significant enrichment of the MAPK signalling pathway, PI3K-Akt signalling pathway, and ECM-receptor interaction in FAPs (Supplementary Fig. 7c); the PI3K-Akt signalling pathway, MAPK signalling pathway, and ECM-receptor interaction in fibroblasts (Supplementary Fig. 7d); the PI3K-Akt signalling pathway, MAPK signalling pathway, cGMP-PKG signalling pathway, and ECM-receptor interaction in PDE4D^+^/PDE7B^+^ subclusters (Supplementary Fig. 7e). These data indicated that the signaling pathways enriched by three different subclusters in FAPs had some similar characteristics.

To further explore the differentiated trajectory of FAPs, we performed a trajectory analysis of FAPs/fibroblasts using Monocle 3. As shown in Fig. 5d, the pseudotime trajectory analysis showed that FAPs could differentiate in two directions, fibroblasts and PDE4D^+^/PDE7B^+^ subclusters with two bifurcations. Interestingly, in the HLW group, more FAPs differentiated into PDE4D^+^/PDE7B^+^ subclusters compared to LLW group. The pseudotemporal heatmap showed gene expression dynamics at Point 2 (Supplementary Fig. 7f).

To identify the adipogenic differentiation of FAPs, we isolated FAPs from pigs and confirmed that FAPs had the capacity to differentiate into adipocytes in vitro (Fig. 5e). Similarly, in 3D cultured FAPs, we also discovered the mature lipid droplets in differentiated FAPs (Fig. 5f). In vivo, we used a glycerol (GLY)-injured model of *Pdgfrα*^*cre-ER*^*/ROSA*^*mTmG*^ mice and sampled TA at 14 DPI (Fig. 5g). We found generated adipocytes mostly expressed *Pdgfrα*^*+*^; however, we also discovered *Pdgfrα*^*-*^ adipocytes in the TA (Fig. 5g). We next isolated IMF cells from the muscle of *Pdgfrα*^*cre-ER*^*/ROSA*^*mTmG*^ mice and induced IMF cell differentiation. Unexpectedly, we found that 43.35% of differentiated lipid droplets expressed *Pdgfrα*^*+*^ while 56.65% expressed *Pdgfrα*^−^ (Fig. 5h), which meant that FAPs are the main source of adipocytes, but not all of them. These results suggested that FAPs could differentiate into IMF cells and contribute to less than 50% of IMF adipocytes.

To explore the other cell origins of IMF, we analysed the transcriptional dynamics in EC, SP, and pericyte clusters. The violin plot showed that EC, SP, and pericyte clusters also expressed preadipocyte-related genes (*CD34*) and adipogenic master genes (*PPARG*) (Fig. 5i, j). Moreover, to further speculate on the potential mechanisms of cellular interaction, we performed cell–cell communication analysis on 9 clusters in muscle nuclei. Interestingly, as shown in Fig. 5k, adipocytes predominantly interacted with SPs, FAPs/fibroblasts, ECs, and pericytes. These data indicated that in addition to FAPs/fibroblasts, SPs, ECs, and pericytes interact to regulate the formation of IMF. However, the detailed function of these cell populations on IMF formation needs to be further explored.

### Transcriptional regulation of lipid deposition in snowflake pork

**Fig. 6:**
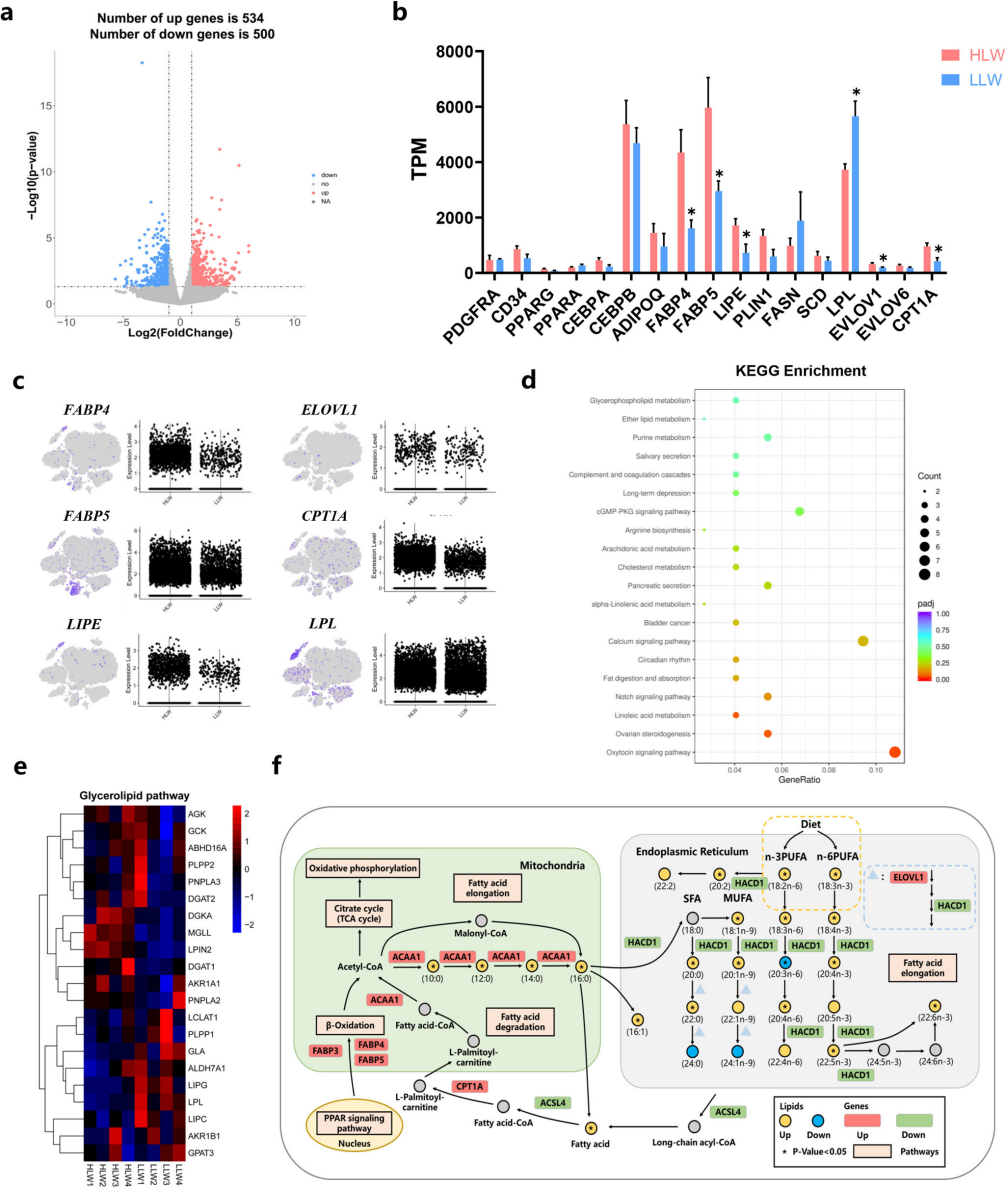
Comparison of gene programs involved in lipid metabolism between the HLW and LLW groups. **a** Volcano plot showing log2-fold changes in exons of RNA-Seq gene bodies in LDM of the HLW and LLW groups (*n* = 4) and the corresponding significance values displayed as log10 (*P* value). The transverse and vertical dotted lines indicate the cut-off value for differential expression (*P* < 0.05 and abs (log2-fold changes) >1). In total, 534 and 500 genes that had upregulated (red) or downregulated (blue) expression levels were identified. **b** TPM values of preadipocyte-related genes, adipogenic master genes, mature adipocyte marker genes, and lipid metabolism-related genes in different groups (*n* = 4). **c** t-SNE and violin plot displaying the expression of differential lipid metabolism-related genes in LDM nuclei. **d** KEGG enrichment analysis of genes in LDM. **e** Heatmap showing the relative expression of glycerolipid pathway**-**related genes derived from the RNA-seq dataset. **f** Selected fatty acid metabolic reactions from KEGG, with indications of quantified lipid classes and acyl chains (circles) and genes (rectangles) significantly regulated compared with LLW group. Statistical analysis was performed using two-tailed Student’s *t*-test.

To further explore the molecular mechanism and associated signalling pathway of snowflake pork, we applied RNA-seq to the LDM of Laiwu pigs. Using a significance level of *P*-value < 0.05 and |log2(fold change) | > 1, we found a total of 1034 DEGs, of which 534 were increased and 500 genes were reduced (Fig. 6a). The expression of these genes related to adipogenesis (*FABP4* and *LIPE*) and lipid metabolism-related genes (*FABP5*, *ELOVL1*, and *CPT1A*) was significantly increased but the expression level of *LPL* was significantly decreased in the HLW group (Fig. 6b). In the snRNA-seq dataset of muscle nuclei, t-SNE and violin plots also showed that the expression levels of *FABP4*, *FABP5*, *LIPE*, *ELOVL1*, and *CPT1A* were relatively increased while that of *LPL* decreased in HLW group (Fig. 6c). However, the expression of the genes related to myogenesis, oxidation-related genes, glycolysis-related genes, and myofiber type transformation-related genes showed no significant difference (Supplementary Fig. 8a).

The KEGG enrichment analysis of the DEGs revealed pronounced changes in glycerophospholipid metabolism, the cGMP-PKG signalling pathway, the calcium signalling pathway, and the Notch signalling pathway (Fig. 6d). The heatmap showed slight differences in genes involved in the glycerolipid pathway (Fig. 6e). In addition, we found that the fatty acid elongation pathway was activated and the expression of *ACAA1*, *CPT1A*, *ELOVL1*, *HACD1* and *ASCL4* was dramatically changed (Fig. 6f). The heatmap showed slight differences in the genes involved in the glycerophospholipid pathway, biosynthesis of the sphingolipid pathway, and fatty acid elongation (Supplementary Fig. 8b–d). These results suggested that the differences in lipid profiles and IMF deposition in LDM might be due to the different expression levels of the genes involved in lipid, fatty acid, and sphingolipid metabolism.

## Discussion

Marbling is one of the important meat quality values, and it affects the sensory properties of pork, including juiciness, tenderness, and flavour^4^. As some cells in skeletal muscle are multinucleated (myofibers) while others (satellite cells, immune cells, fibroblasts, and adipocytes) are mononuclear, it has been technically challenging to explore potential functional distinctions and transcriptional heterogeneity in skeletal muscle. SnRNA-seq is a new high-resolution transcriptome method and is able to assay both mononuclear and multinucleated cells together^11^. In this study, we revealed the different cell type populations, gene expression profiles, and potential mechanisms of snowflake pork by using snRNA-seq combined with lipidome and RNA-seq.

Fat deposition and lipid composition in skeletal muscle are affected by a variety of factors. Previous studies have found that breed is an important factor to affect lipid composition in skeletal muscle of pigs, which is manifested in Laiwu pigs contained more TGs than Yorkshire pigs^19^ and Luchuan pigs had higher TGs and DGs compared to Duroc pigs^20^. Also, abundant TGs, phosphatidylinositols and phosphatidylserines were found to be upregulated in LDM of Xidu black pigs with higher IMF contents^21^. Besides, our previous study has found dietary conjugated linoleic acid supplement could induce lipid dynamics in muscle of Heigai pigs^22^. In our Laiwu pig model, we found that the overall composition of lipid classes had significant heterogeneity. A high IMF content is always accompanied by an increase in glycerolipids and sphingolipids. Previous studies have clarified that adipose tissue has much more glycerolipids than skeletal muscle^23^. In humans with insulin resistance independent of obesity, the sphingolipid metabolism gene pathway was upregulated in muscle^24^. Sphingolipids regulate a plethora of cell biological processes including growth regulation, cell migration, adhesion, apoptosis, senescence and inflammatory responses. They are related to diseases such as cancers, metabolic disorders, immune function, cardiovascular disorders and skin integrity at the tissue and organismal levels^25^. In muscle, intramuscular lipid droplets primarily comprise TGs^26^. In our study, we found higher contents of TGs and Cers in the HLW group. In addition, snRNA-seq and RNA-seq results showed that the expression of fatty acid and sphingolipid metabolism-related genes was significantly changed. Another local Chinese pig species, Luchuan pigs, had upregulated contents of TGs and Cers compared with Duroc pigs^20^. Similarly, in GLY-injected mice, the contents of total TGs and Cers were also significantly increased in muscle^9^. However, previous studies found that obesity induces upregulated levels of Cers and that increased Cers correlate with the pathogenesis of metabolic diseases^27^. Except for TGs and Cers, we also found that the content of StEs was also higher in the HLW group. It has been reported that stigmasterol could protect against high-fat Western diet-induced nonalcoholic fatty liver disease^28^. Besides, we found there is a reduced tendency in glycerophospholipids, including PEs, PSs, and PCs. Previous study has found adipose tissue had a lower concentration of glycerophospholipids, including PGs, PSs, PEs, and PCs^23^. However, there is still a blank in the association between glycerophospholipids and meat quality. These results indicated that lipids may serve as new biomarkers for marbling formation and that IMF deposition is always accompanied by the increased contents of TGs and Cers but decreased contents of glycerophospholipids in muscle. However, the physiological functions of these lipids need to be further explored.

Skeletal muscle is the largest tissue in the human body and comprises many cell types. The synergistic action of these single cells together determines the biological function of skeletal muscle. Recently, an increasing number of studies have focused on exploring the cell sources and heterogeneity of skeletal muscle in human and animal models by using high throughput single-cell and subcellular resolution transcriptomics such as scRNA-seq^29,30^ and ST^10,31^. However, it is challenging to assay larger cell types by scRNA-seq and ST due to the size of myotubes and myofibers. Another new high-resolution transcriptome method, snRNA-seq, can profile the transcriptomes of mature muscle cells such as myotubes or myofibers at the whole cell or nucleus level^16^. Here, we used snRNA-seq to analyse the dynamics of cell populations in muscle of pigs. Although we have reduced the problem of cell debris affecting the number of captured nuclei through CellBender, we found there is still a difference in collected nuclei between two groups. We identified 9 cell populations in the skeletal muscle of Laiwu pigs, including myofibers, FAPs/fibroblasts, MuSCs, ECs, pericytes, myeloid-derived cells, immune cells, adipocytes, and SPs. HLW groups had the lower myofiber nuclei and the higher adipocytes nuclei, MuSCs nuclei and FAPs nuclei. FAPs are the main source of IMF^32^. and previous study found some MuSCs can differentiate into adipogenic cells^33^. Besides, adipocytes are easier to digest than muscle, hence, we think the difference in collected nuclei may result from the more resistant to enzyme digestion of LLW groups. Likewise, Dos Santos et al. and Petrany *et al*. identified many cell types, including SCs, FAPs, tenocytes, endothelial cells, smooth muscle cells, pericytes, B/T cells immune cells, and adipocytes in adult mouse skeletal muscle by snRNA-seq^16,34^. In a mouse model of DMD caused by deletion of exon 51 of the dystrophin gene, Chemello et al. identified three clusters of nuclei related to skeletal muscle regeneration including cluster MuSC, cluster Myog, and cluster RegMyon^11^.

Skeletal muscle comprises slow and fast myofibers which are classified by MYH (myosin heavy chain) expression^35,36^. Four major myonuclei types with distinct myosin compositions have been identified in the skeletal muscle of mice, including slow myofibers with type I myonuclei (*Myh7*), fast myofibers with type IIa myonuclei (*Myh2*), type IIx myonuclei (*Myh1*), and type IIb myonuclei (*Myh4*)^16,34^. In our study, we identified four muscle fiber type in pigs, including type I myonuclei (*MYH7*) and type IIa myonuclei (*MYH2*), type IIx myonuclei (*MYH1*), and type IIb myonuclei (*MYH4*). We found that the HLW group had a relatively higher content of type IIa myonuclei but a relatively lower content of type IIb myonuclei. Previous study has found *MyHC IIa* expression was negatively related to muscle fiber diameter but *MyHC IIb* expression was postively related to muscle fiber diameter^37^. Besides, the diameter of slow myofiber was always smaller than the diameter of fast myofiber which could explain why the HLW group had a smaller muscle fiber diameter. A previous study found that slow-twitch oxidative type I myonuclei have higher activities in myoglobin and mitochondrial oxidative metabolic enzymes while fast-twitch glycolytic type II myonuclei have higher activities in glycogen and glycolytic enzymes^13,36^. This is consistent with our findings that the expression of oxidation-related genes was relatively upregulated while glycolysis-related genes were relatively downregulated in the HLW group. Overall, our results indicate that the skeletal muscle of pigs has great heterogeneity and that muscle fiber types are associated with muscle fiber diameter and IMF deposition.

Marbling is one of the important indexes to evaluate the pork quality and is associated with flavour, tenderness, and juiciness. Interestingly, we found that compared with the LLW group, the HLW group had a higher proportion of DGAT2^+^/SCD^+^ subclusters and FABP5^+^/SIAH1^+^ subclusters in adipocytes clusters. DGAT1 and DGAT2 are important for fatty acid esterification into TGs^38^ and are always accompanied by increasing TG levels^39^. A previous study found that during adipogenesis in white preadipocytes, the expression of *DGAT2*, *SCD*, and *FABP5* was significantly higher in Cluster 1, as detected by snRNA-seq and scRNA-seq^40^. These results indicated that DGAT2^+^/SCD^+^ subclusters and FABP5^+^/SIAH1^+^ subclusters may be the key differentiated cell populations between the HLW and LLW groups.

Uezumi et al. verified that a subpopulation of PDGFRα^+^ or CD140α^+^ progenitor cells is the main source of intramuscular adipocytes during muscle regeneration in mice, and they are defined as FAPs^32^. However, there is very little research on FAPs in pigs. In our study, we identified three subclusters in FAPs/fibroblasts, including FAPs, fibroblasts, and PDE4D^+^/PDE7B^+^ subclusters. Interestingly, we found that PDE4D^+^/PDE7B^+^ subclusters are similar to one of the subclusters in adipocytes and trajectory analysis indicated that in the HLW group, more FAPs differentiated into PDE4D^+^/PDE7B^+^ subclusters compared to LLW group. In addition, we confirmed that FAPs have the capacity to differentiate into adipocytes in vitro. Hence, we speculated that PDE4D^+^/PDE7B^+^ subclusters might be the main cell populations in the process of preadipocyte differentiation in differential IMF deposition. However, further studies need to verify our hypothesis. Unexpectedly, although FAPs contribute to IMF formation, we found that less than 50% of adipocytes are derived from FAPs, suggesting that other types of cells may also be involved in marbling formation. However, the proportion of FAPs during the regeneration phase and the effects of injection time and sustainability of tamoxifen on cell proportion of differentiated IMF cells should be considered in further studies.

Notably, we observed that the expression of preadipocyte-related genes was higher in EC, SP, and pericyte populations of the HLW group and adipocytes mainly interacted with SPs, FAPs/fibroblasts, ECs, and pericytes in turn. Existing evidence supports that some ECs, SPs, and pericytes have the potential for adipogenic differentiation in vitro^41–43^. Overall, our results indicate that the heterogeneity of adipocytes and adipogenic cell populations such as FAPs, ECs, SPs, and pericytes is the potential regulatory mechanism of IMF deposition. However, future work should reveal the functional significance of different cell populations during IMF deposition.

Marbling formation is a complex process, and the regulatory mechanism of IMF deposition involves genes related to lipid metabolism signaling pathways^44^. In our study, we found that the expression of *FABP4*, *FABP5*, *LIPE*, *ELOVL1*, and *CPT1A* was positively associated with IMF deposition, but *LPL* expression was negatively associated with IMF deposition. Lipoprotein lipase (LPL) is an enzyme that catalyzes the hydrolysis of protein-linked TGs and plays a key role in TG metabolism. In Italian Large White pigs, *LIPE* and *LPL* gene expression has been reported to be related to IMF deposition^45^. However, further studies are required to explore the special regulatory mechanism of *LPL* in IMF deposition. In addition, we discovered that *FABP5* was highly expressed in one of the adipocytes subclusters. FABP5, together with FABP4, is the main FA-binding protein in adipose tissue^46^. Previous studies demonstrated that septin 11 could bind to FABP5 to regulate adipocyte metabolism in 3T3-L1 cells^47^. *ELOVL1* has been suggested to be involved in the elongation of SFAs and MUFAs and to regulate lipid metabolism^48^. For signalling pathways, we found that DEGs were enriched in glycerophospholipid metabolism, the cGMP-PKG signalling pathway, the calcium signalling pathway, and the Notch signalling pathway. Consistent with our findings, Xu et al. found that the MAPK signalling pathway and calcium signalling pathway were significantly associated with IMF deposition^44^. Notch signalling plays an important role in regulating metabolic disorders and participates in beige adipocyte formation, adipocyte dedifferentiation and function^49,50^. We also found that the significant changes in genes in the glycerolipid pathway resulted in the alteration of TG contents. Likewise, in our previous GLY-induced IMF deposition mice, the glycerolipid pathway and glycerophospholipid pathway were also changed^9^. However, the functions of these signalling pathways in IMF deposition need to be further explored.

In conclusion, we provide detailed insights into the cellular and molecular signatures of marbling formation by using a Laiwu pig model with a high IMF content. We identified 3 subpopulations (PDE4D^+^/PDE7B^+^, DGAT2^+^/SCD^+^ and FABP5^+^/SIAH1^+^ cells) of adipocytes and found that FAPs could differentiate into IMF cells and contribute to less than 50% of adipocytes. Our data also described different genes involved in lipid metabolism and fatty acid elongation. Our findings could provide a foundation for developing new strategies to increase IMF deposition and the lipo-nutritional quality of snowflake pork.

## Methods

### Animals and samples

All the procedures involving mice were approved by Zhejiang University Animal Care and Use Committee. Laiwu pigs from the original Laiwu pig breeding farm and Laiwu Pig Breeding Co., LTD were used for the study. Laiwu pigs were fed full-price compound feed with the same nutritional content according to their age and provided with a 12 h light/dark cycle and free access to water and food. Laiwu pigs were slaughtered at the same slaughterhouse when they weighed approximately 100 kg and were humanely sacrificed after fasting for 12–16 h. Samples of longissimus dorsi muscle (LDM) were obtained from the 3rd to 11th rib on the right side of the carcass for meat quality measurement. Carcass traits and meat quality were measured as previous describrd^22^. and the IMF content are shown as the weights of fat or protein per 100 g of freeze-dried LDM (g per 100 g). Based on the determination of IMF content in Laiwu pigs, we chose six Laiwu pigs with higher IMF content and six Laiwu pigs with lower IMF content then divided them into two groups: the HLW group (high IMF content) and the LLW group (low IMF content). Approximately 200 g of LDM was rapidly collected, frozen in liquid nitrogen immediately, and subsequently stored at −80 °C for lipidomics determination. The two most representative samples from each group were selected for later snRNA-seq and four samples from each group were selected for RNA-seq analyses. A block of LDM was removed from the body and placed into a fixative solution for haematoxylin-eosin (H&E) staining.

For the lineage tracing experiment, C57BL6/J mice were housed under standard laboratory conditions with a 12 h light/dark cycle and free access to water and standard rodent chow. *Pdgfrα-Cre*^*ER*^ (stock no. 018280) and *ROSA*^*mT/mG*^ (stock no. 007676) mice were purchased from Jackson Laboratory (Bar Harbor, ME, USA), and *Pdgfrα*^*cre-ER*^*/ROSA*^*mTmG*^ mice were generated. PCR genotyping was performed using protocols described by the supplier. All mice were anaesthetized and injected with 50 μL 50% GLY into the tibialis anterior (TA) muscle after being fed standard rodent chow food for 14 days as previously described^9^. At 14 days postinjection (DPI), the TA samples were carefully sampled, placed into a mould, buried in OCT, frozen immediately in liquid nitrogen, and stored at −80 °C for subsequent sectioning. The freezing slicer setting program was temperature −20 °C and section thickness 8 μm.

### Hematoxylin-eosin (H&E) staining

LDM from Laiwu pigs were fixed in 4% paraformaldehyde for 24 h at room temperature. Then, the tissues were removed from the fixative solution for paraffin embedding and microtome sectioning. Paraffin sections were dewaxed in water: the sections were placed in xylene I for 10 min, xylene II for 10 min, anhydrous ethanol I for 5 min, anhydrous ethanol II for 5 min, 95% alcohol for 5 min, 80% alcohol for 5 min, and 70% alcohol for 5 min and washed in tap water. The sections were stained with haematoxylin for 15 min and eosin solution for 3–5 min and after anhydrous ethanol I for 5 min, anhydrous ethanol II for 5 min, anhydrous ethanol III for 5 min, dimethyl I for 5 min, and xylene II for 5 min; sealed with neutral gum; and mounted for microscopic examination.

### Immunofluorescence

The dewaxed sections were immersed in sodium citrate and placed in a microwave oven for 15 min for antigen retrieval. After the sections cooled to room temperature, they were fixed in 4% paraformaldehyde for 10 min, permeated with 0.5% Triton-X100 for 10 min and blocked with blocking buffer (PBS, 5% goat serum and 2% BSA) for 1 h. Then, the sections were incubated with Perilipin (Abcam, ab16667, 1:500) and MF20 (Developmental Studies Hybridoma Bank, 1:50) primary antibodies diluted in blocking buffer overnight. After washing with PBS three times for 10 min, the samples were incubated with secondary antibodies and DAPI for 6 min at room temperature. After sealing with glycerol, fluorescent images were captured as single-channel greyscale images using a Leica DM 6000B fluorescence microscope with a ×20 objective (NA 0.70).

### Lipidomic assay

200 μL cold water and 20 μL lipid internal standard mixture were added to 60 mg LDM tissue, then the samples were homogenized on a MP homogenizer at 4 °C (24 × 2, 6.0 M/S, 60 s, twice). Following this, 800 μL cold methyl tert-butyl ether and 240 μL methanol were added to the samples and vortexed for 30 s, sonicated at 4 °C for 20 min and stand for 30 min, then centrifuged (14000 *g* for 15 min at 10 °C) to extract lipids. The upper organic layer was dried in a vacuum centrifuge. Prior to analysis, lipid extracts were resuspended in 200 μL of isopropanol acetonitrile 9:1 (v/v). In brief, lipids were separated on a Waters ACQUITY PREMIER CSH C18 Column (1.7 μm, 2.1 × 100 mm). MS detection was performed using a Thermo Scientific™ Q Exactive mass spectrometer mass spectrometer, equipped with an ESI ion source. Data were acquired in both positive and negative ion modes, respectively. Data-dependent acquisition methods were used for MS/MS analyses of lipidome. Lipidsearch 4.0 software was used for peak detection and annotation of lipids or internal standards.

### Longissimus dorsi muscle nuclei isolation

Longissimus dorsi muscle nuclei were isolated with Nuclei EZ Lysis buffer (NUC-101; Sigma‒Aldrich) supplemented with protease inhibitor (5892791001; Roche) and RNase inhibitor (N2615; Promega and AM2696; Life Technologies). Samples were cut into 2 mm pieces and homogenized using a Dounce homogenizer (885302-0002; Kimble Chase) in 2 ml of ice-cold Nuclei EZ Lysis buffer, and they were incubated on ice for 5 min with an additional 2 ml of lysis buffer. The homogenate was filtered through a 40 mm cell strainer (43-50040-51; pluriSelect) and then centrifuged at 500 × *g* for 5 min at 4 °C. The pellet was resuspended and washed with 4 ml of the buffer and then incubated on ice for 5 min. After another centrifugation, the pellet was resuspended in Nuclei Suspension Buffer (PBS, 0.07% BSA, and 0.1% RNase inhibitor), filtered through a 20 μm cell strainer (43-50020-50; pluriSelect), and counted.

### 10X Genomics Chromium library and sequencing

Single-cell suspensions were loaded onto 10X Genomics Chromium to capture 5000 single cells according to the manufacturer’s instructions for the 10X Genomics Chromium Single-Cell 3’ kit (V3). The following cDNA amplification and library construction steps were performed according to the standard protocol. Libraries were sequenced on an Illumina NovaSeq 6000 sequencing system (paired-end multiplexing run, 150 bp) by LC-Bio Technology Co., Ltd. (Hangzhou, China) at a minimum depth of 20,000 reads per cell.

### Bioinformatics analysis

The sequencing results were demultiplexed and converted to FASTQ format using Illumina bcl2fastq software (version 2.20). Sample demultiplexing, barcode processing and single-cell 3’ gene counting were performed using the Cell Ranger pipeline (https://support.10xgenomics.com/single-cell-gene-expression/software/overview/welcome, version 6.1.2), and snRNA-seq data were aligned to Ensmebl Sus scrofa genome v96 as the reference genome. A total of 56108 single cells captured from 2 HLW pigs and 2 LLW pigs were processed using 10X Genomics Chromium Single Cell 3’ Solution. The Cell Ranger output was loaded into Seurat (version 4.1.0) for dimensional reduction, clustering, and analysis of snRNA-seq data. Overall, 47273 cells passed the quality control threshold: the number of genes expressed per cell >500 and the percent of mitochondrial DNA-derived gene expression <25%. The DoubletFinder package (version 2.0.3) was used to remove doublets. We also performed batch correction using Harmony for data integration between samples.

To visualize the data, we further reduced the dimensionality of all 47273 cells using Seurat and used t-SNE to project the cells into 2D space. The steps include the following: 1. The LogNormalize method of the “Normalization” function of Seurat software was used to calculate the expression value of genes. 2. Principal component analysis (PCA) was performed using the normalized expression value. Among the PCs, the top 10 PCs were used to perform clustering and t-SNE analysis. 3. To find clusters, the weighted shared nearest neighbour (SNN) graph-based clustering method was selected. Marker genes for each cluster were identified with the Wilcoxon rank-sum test with default parameters via the FindAllMarkers function in Seurat. This selects marker genes that are expressed in more than 10% of the cells in a cluster, *P* value ≤ 0.01, and average log2(fold change)≥0.26.

### Pseudotime analysis and cell–cell communication analysis

To model differentiation trajectories, we performed trajectory analysis using Monocle 2 (http://cole-trapnell-lab.github.io/monocle-release/docs, version 2.22.0). For cell communication analysis, we used CellPhoneDB (https://www.cellphonedb.org, version 3.1.0) to further speculate on the underlying cellular interaction mechanisms.

### Primary cell isolation, magnetic cell sorting and cell culture

Primary IMF cells were isolated from hind limb skeletal muscle in *Pdgfrα*^*cre*^*/ROSA*^*mTmG*^ mice. Muscles were minced and digested in a type I collagenase and dispase B mixture (2 mg/mL collagenase, 1.5 mg/mL dispase and 2.5 μL/mL 1 M CaCl_2_ in PBS) for 60 min at 37 °C. The cells were then filtered from debris through 70 μm filters, centrifuged at 600 × *g* for 6 min and cultured in growth media (F-10 Ham’s medium supplemented with 20% foetal bovine serum, 10 ng/ml basic fibroblast growth factor and 2% penicillin-streptomycin) on collagen-coated dishes. The medium was refreshed every two days. For magnetic cell sorting, primary FAPs were isolated from LDM in 3-day-old piglets. Muscles were minced and digested in 0.2% type I collagenase at 37 °C for 60 min. The cells were then filtered from debris through sterilized gauze and centrifuged at 500 × *g* for 6 min. After the supernatant was removed, red blood cell lysate (AR1118, Boster Biological Technology Co., Ltd., China) was added to split at 4 °C for 5 min. Then, the cells were incubated at room temperature for 15 min with a Dead Cell Removal Kit (130-090-101, Miltenyi Biotec, Bergisch Gladbach, Germany) and centrifuged at 400 × *g* for 5 min. CD140a antibody (CD140a antibody, anti-mouse, 130-101-905, Miltenyi Biotec, Bergisch Gladbach, Germany) was added, followed by incubation at 4 °C for 5 min and centrifugation at 400 × *g* for 4 min. Then, 20 μL antibiotin microbeads (130-090-485, Miltenyi Biotec, Bergisch Gladbach, Germany) were added, followed by incubation at 4 °C for 15 min and centrifugation at 400 × *g* for 4 min. After the magnetic column, the cells on the adsorption column were PDGFRα^+^ cells. For adipogenic differentiation and 3D cluture, the cells were induced to differentiate when they reached 90% confluence by induction medium (DMEM, 10% FBS, 10 μg/ml insulin, 1 μM dexamethasone (DEXA) and 500 μM 3-isobutylmethylxanthine (IBMX)) for 3 days upon confluence and then differentiated in differentiation medium (DMEM, 10% FBS, 10 μg/ml insulin) for 2 days until the adipocytes matured.

### Total RNA extraction and qRT-PCR

Total RNA was extracted from FAPs using TRIzol Reagent (Yeasen, China), and the purity and concentration of total RNA were measured by a spectrophotometer (NanoDrop 2000; Thermo Fisher Scientific) at 260 and 280 nm. Random primers and a ReverAid First Strand cDNA Synthesis Kit (Thermo Fisher) were used to reverse RNA samples. qPCR was performed with an Applied Biosystems StepOnePlus Real-Time PCR System using Hieff qPCR SYBR® Green Master Mix (Yeasen) and gene-specific primers (Supplementary Table 1). The 2^−ΔΔCT^ method was used to analyse the relative changes in gene expression normalized against 18 S ribosomal RNA as an internal control.

### RNA-seq analysis

Total RNA was extracted from the LDM of Laiwu pigs by the TRIzol method. Sequencing libraries were generated from RNA samples with a total amount of more than 3 µg and RIN value greater than 8.0 using the NEBNext® Ultra™ RNA Library Prep Kit for Illumina. The qualified libraries were sequenced using the Illumina HiSeq sequencing platform at 150 bp. Raw reads were filtered to remove reads with adapters, reads with undetermined base information (N) content greater than 1% and reads with Qphred ≤20 accounting for more than 50% of the entire read length. Clean reads were mapped to the pig reference genome (Sscrofa11.1) and gene model annotation file (Ensembl Genes 103) used by HISAT2 (version 2.0.5). The number of reads mapped to each gene was calculated using featureCounts (1.5.0-p3), and the FPKM of each gene was calculated according to its length. Gene expression values of the transcripts were computed by StringTie (version 1.3.3b). Differentially expressed genes (DEGs) were identified between the two groups by using the package DESeq2 (version 1.12.4). Genes with a *P* value < 0.05 were considered significant DEGs.

### Pathway enrichment analysis

Gene Ontology (GO) and Kyoto Encyclopedia of Genes and Genomes (KEGG) enrichment analyses were used to identify which DEGs were significantly enriched in GO terms or metabolic pathways. GO terms and KEGG pathway analyses using the hypergeometric test were performed to identify significantly enriched metabolic pathways or signal transduction pathways enriched in DEGs. GO terms and KEGG pathways with false discovery rates *P* < 0.05 were considered significantly different.

### Statistical analysis

Experimental data are presented as the means±standard errors of the means (SEM) from at least three independent experiments. GraphPad (Prism 8.3.0) and R software (version 4.1.3) were used for data analyses and data visualization. Comparisons were made by unpaired two-tailed Student’s *t* tests. Differences were considered significant at *P* < 0.05.

## Supplementary information

Supplementary Information

## Data Availability

The raw snRNA-seq data and bulk RNA-seq data reported in this paper have been deposited in the Genome Sequence Archive (Genomics, Proteomics & Bioinformatics 2021) in National Genomics Data Center (Nucleic Acids Res 2022), China National Center for Bioinformation / Beijing Institute of Genomics, Chinese Academy of Sciences (GSA: CRA011059 and CRA011069) that are publicly accessible at https://ngdc.cncb.ac.cn/gsa.
