# Supplementary material for: Single-nucleus and bulk RNA sequencing reveal cellular and transcriptional mechanisms underlying lipid dynamics in high marbled pork
Source: NPJ Sci Food. 2023 Jun 2;7:23. doi: 10.1038/s41538-023-00203-4 (PMC10238404; doi:10.1038/s41538-023-00203-4)
Supplement: Supplementary file 1 — Supplementary Information [file 41538_2023_203_MOESM1_ESM.pdf]

Supplementary Table 1. The primer sequence of qPCR

Primer name	Sequence (5'-3')
18s-F	CCCACGGAATCGAGAAAGAG
18s-R	TTGACGGAAGGGCACCA
FABP4-F	TGGAAACTTGTCTCCAGTG
FABP4-R	GGTACTTTCTGATCTAATGGTG
LEPTIN-F	GGCCCTATCTGTCCTACGTTGAAG
LEPTIN-R	TGGAAGGCAGACTGGTGAGGAT
PLIN1-F	CCCCCTGGTGGCGTCTGTAT
PLIN1-R	ACTGGAGGGCCGGTATCTTTTCT

Supplementary Figure 1

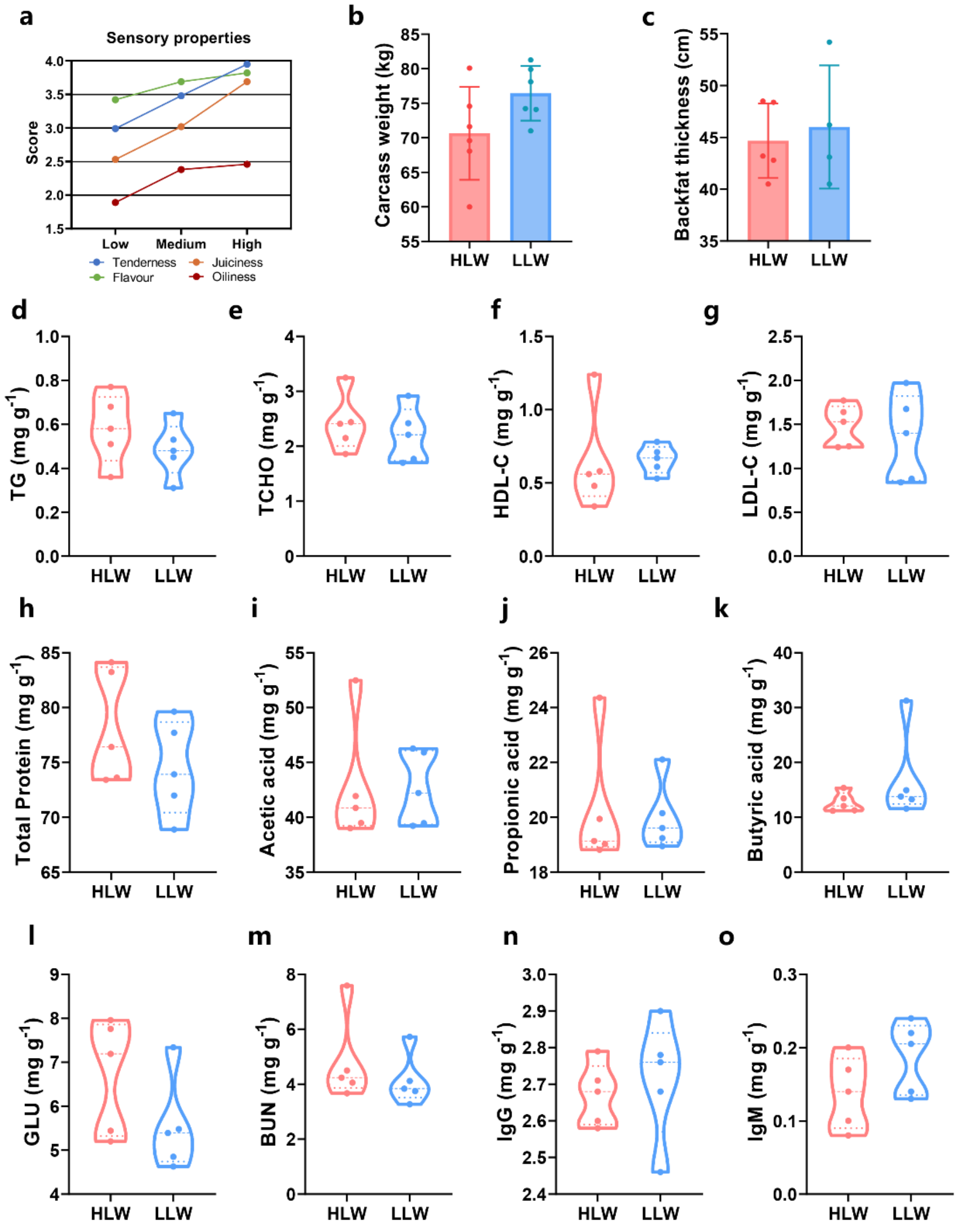

Supplementary Figure 1. The importance of marbling and carcass traits and biochemical indexes in serum of Laiwu pigs. (a) Marbling affects sensory properties. **(b)** Carcass weight in the HLW and LLW groups (n=6). **(c)** Backfat thickness in the HLW and LLW groups (n=6). **(d-o)** Biochemical indexes in serum (TG, TCHO, HDL-C, LDL-C, total protein, acetic acid, propionic acid, butyric acid, GLU, BUN, IgG and IgM) of different groups (n=5). Error bars represent SEM, two-tailed Student's t test.

Supplementary Figure 2

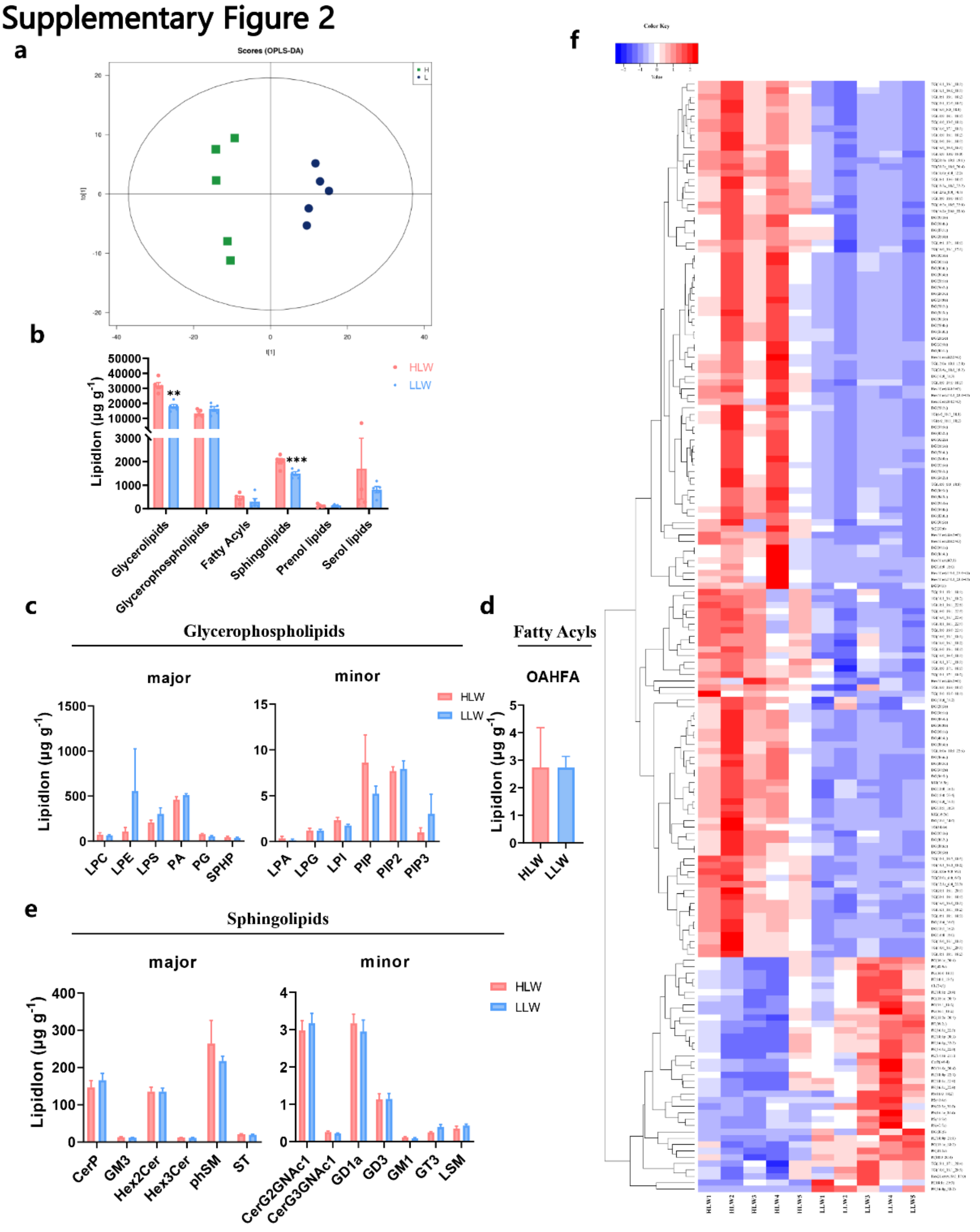

Supplementary Figure 2. Changes of the overall lipid composition and distribution in the HLW and LLW groups. (a) OPLS-DA plot of differentially lipids in different groups. **(b)** The concentration of lipid species in different groups. **(c-e)** The contents of glycerophospholipids **(c)**, fatty acyls **(d)**, sphingolipids **(e)** in LDM from the HLW and LLW groups (n = 5). **(f)** Heatmap of differentially lipid species in different groups. Error bars represent SEM, $^{**}P < 0.01$, $^{***}P < 0.001$, two-tailed Student's t-test.

Supplementary Figure 3

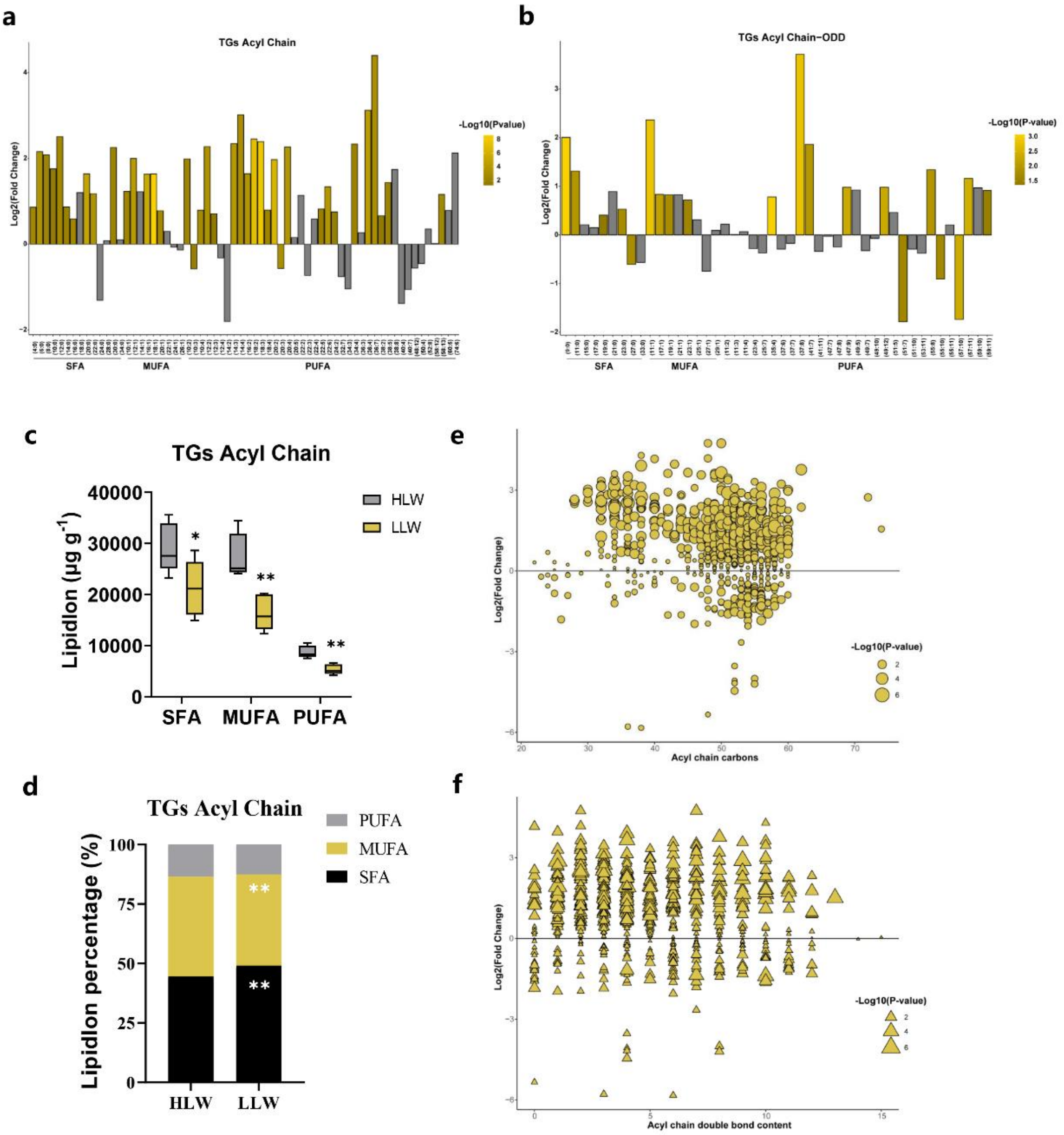

Supplementary Figure 3. Comparison of TGs acyl chain composition between the HLW and LLW groups. (a) The total intensity fold changes of individual fatty-acyl chains associated with TGs sorted by degree of saturation. **(b)** The total intensity fold changes of odd-numbered fatty acyl chains associated with TGs. Odd, odd-numbered fatty acyls. The transparency of each bar is proportional to the significance values, which are displayed as -Log10 (*P*-value). The grey bars indicate those with $P > 0.05$.

(c) TGs acyl chain content at different saturation levels. Box indicates IQR; whisker indicates min or max; plus shows mean. **(d)** TGs acyl chain percentage at different saturation levels. **(e)** TGs with different numbers of carbon atoms. **(f)** TGs with different numbers of double bonds content. SFA, saturated fatty acyls; MUFA, monounsaturated fatty acyls; PUFA, polyunsaturated fatty acyls. n = 5. Error bars represent SEM. $*P < 0.05$, $**P < 0.01$, two-tailed Student's t-test.

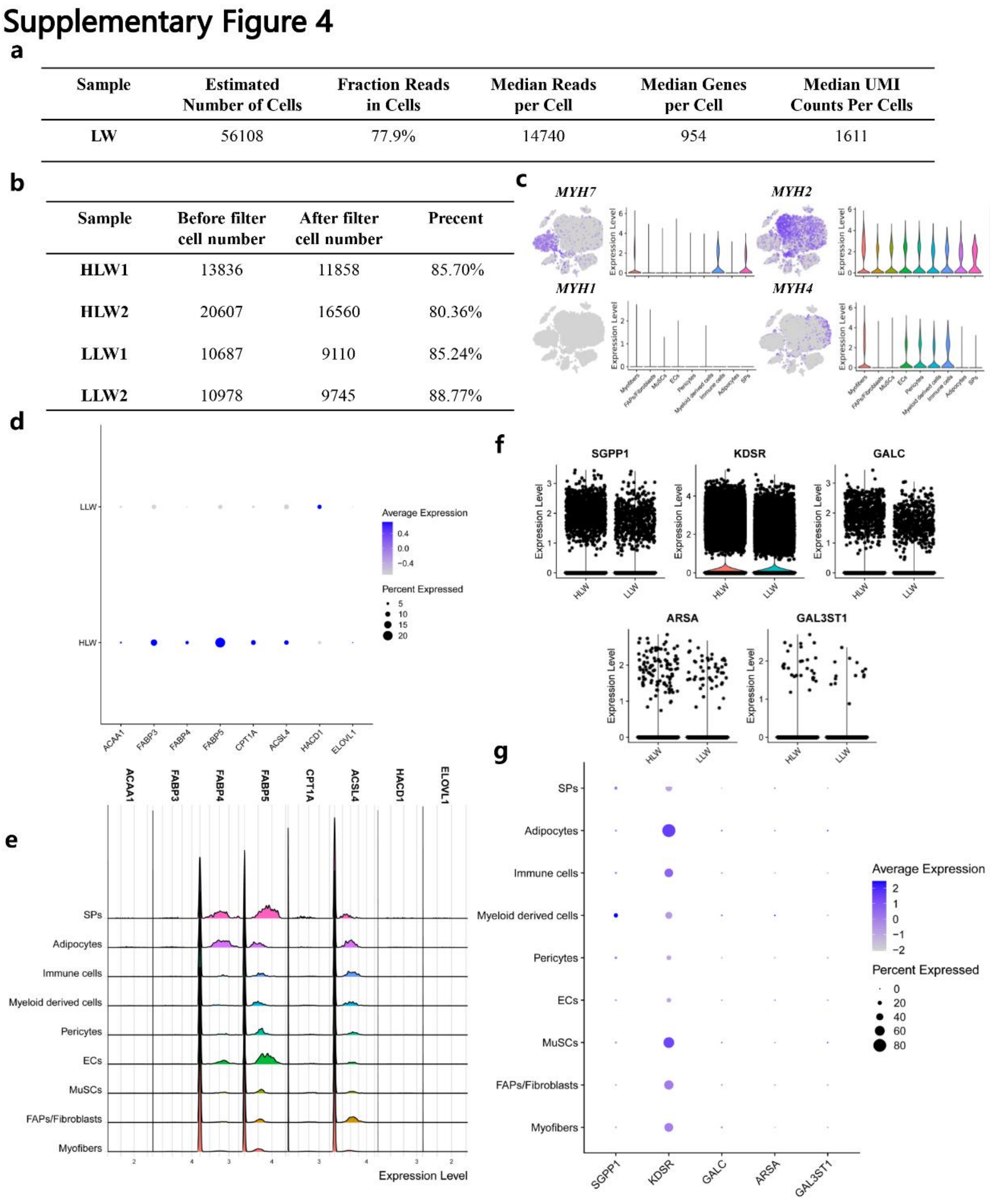

Supplementary Figure 4

a

Sample	Estimated Number of Cells	Fraction Reads in Cells	Median Reads per Cell	Median Genes per Cell	Median UMI Counts Per Cells
LW	56108	77.9%	14740	954	1611

b

Sample	Before filter cell number	After filter cell number	Precent
HLW1	13836	11858	85.70%
HLW2	20607	16560	80.36%
LLW1	10687	9110	85.24%
LLW2	10978	9745	88.77%

Supplementary Figure 4. The expression of fatty acid and sphingolipid metabolism-related genes in cell populations and different groups. (a) The results obtained from Cell Ranger analyses. **(b)** Cell number of snRNA-seq datasets before or after filter from each sample. **(c)** tSNE and violin plot displaying the expression of myosin heavy chain genes genes for each cluster of LDM nuclei. **(d)** Dot plot showing the expression of fatty acid metabolism-related genes in the HLW and LLW groups of

LDM nuclei. **(e)** Ridge plot displaying the expression of fatty acid metabolism-related genes in different cell clusters of LDM nuclei. **(f)** Violin plot showing the expression of sphingolipid metabolism-related genes in the HLW and LLW groups of LDM nuclei. **(g)** Dot plot displaying the expression of sphingolipid metabolism-related genes in different cell clusters of LDM nuclei.

Supplementary Figure 5

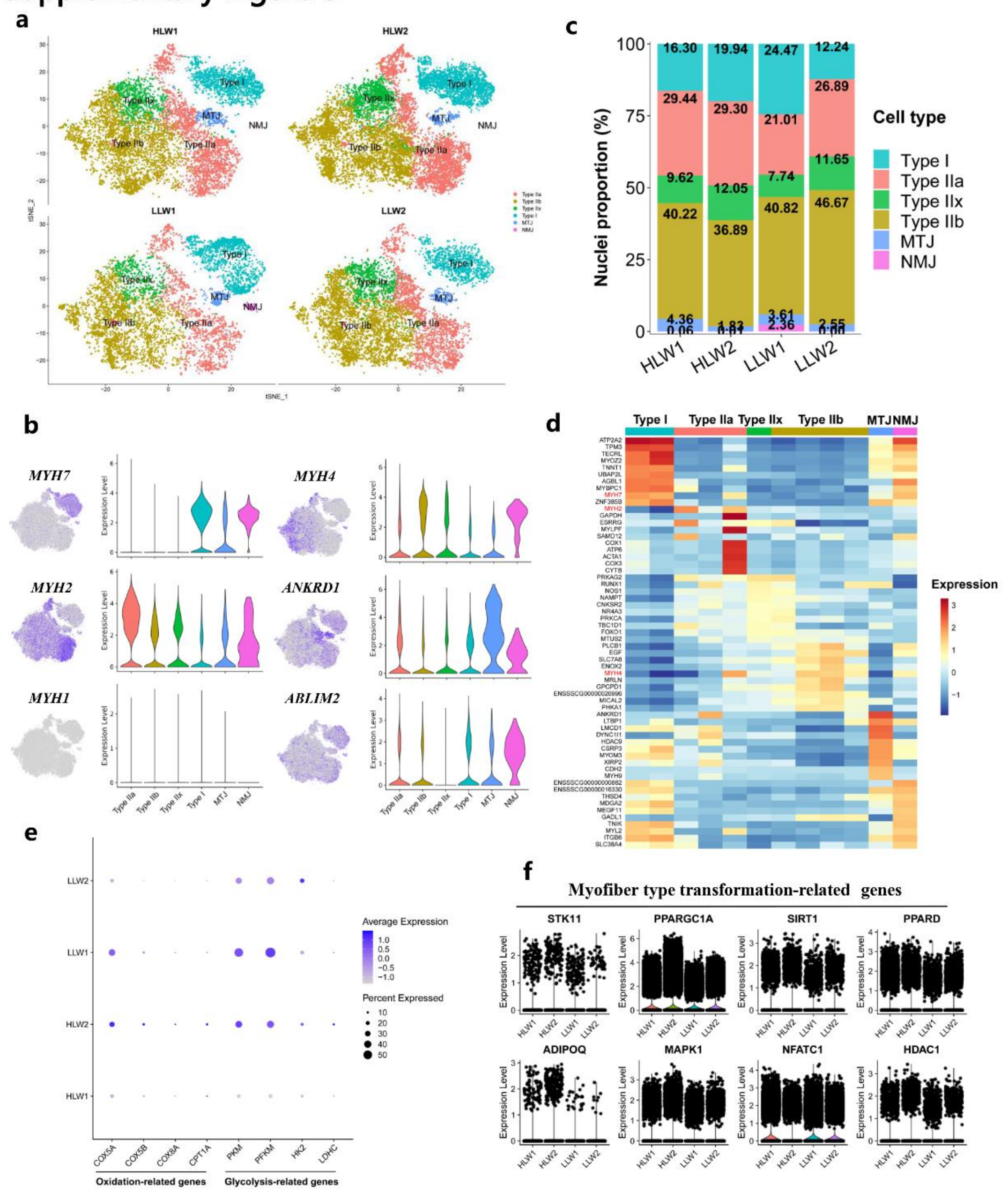

Supplementary Figure 5. Transcriptional heterogeneity in myofibers nuclei. (a) tSNE plot showing six subclusters of the isolated single nuclei from the HLW and LLW muscles. **(b)** tSNE and violin plot displaying the expression of selected marker genes for each subcluster of nuclei. **(c)** Nuclei proportion in each subcluster in the HLW and LLW groups. Each cluster is colour-coded. **(d)** Heatmap representing the top 10 most differently expressed genes between cell subclusters identified. **(e)** Dot plot showing the expression of oxidation-related genes (*COX5A*, *COX5B*, *COX8A*, and *CPT1A*), glycolysis-related genes (*PKM*, *PFKM*, and *HK2*) in different group. **(f)** Violin plot showing the expression of myofiber type transformation-related genes (*STK11*, *PPARGC1A*, *SIRT1*, *PPARD*, *ADIPOQ*, *MAPK1*, *NFATC1*, and *HDAC1*) in different group. Type I, type I myonuclei; Type IIa, type IIa myonuclei; Type IIx, type IIx myonuclei; Type IIb, type IIb myonuclei; MTJ, myotendinous junction nuclei; NMJ, neuromuscular junction nuclei.

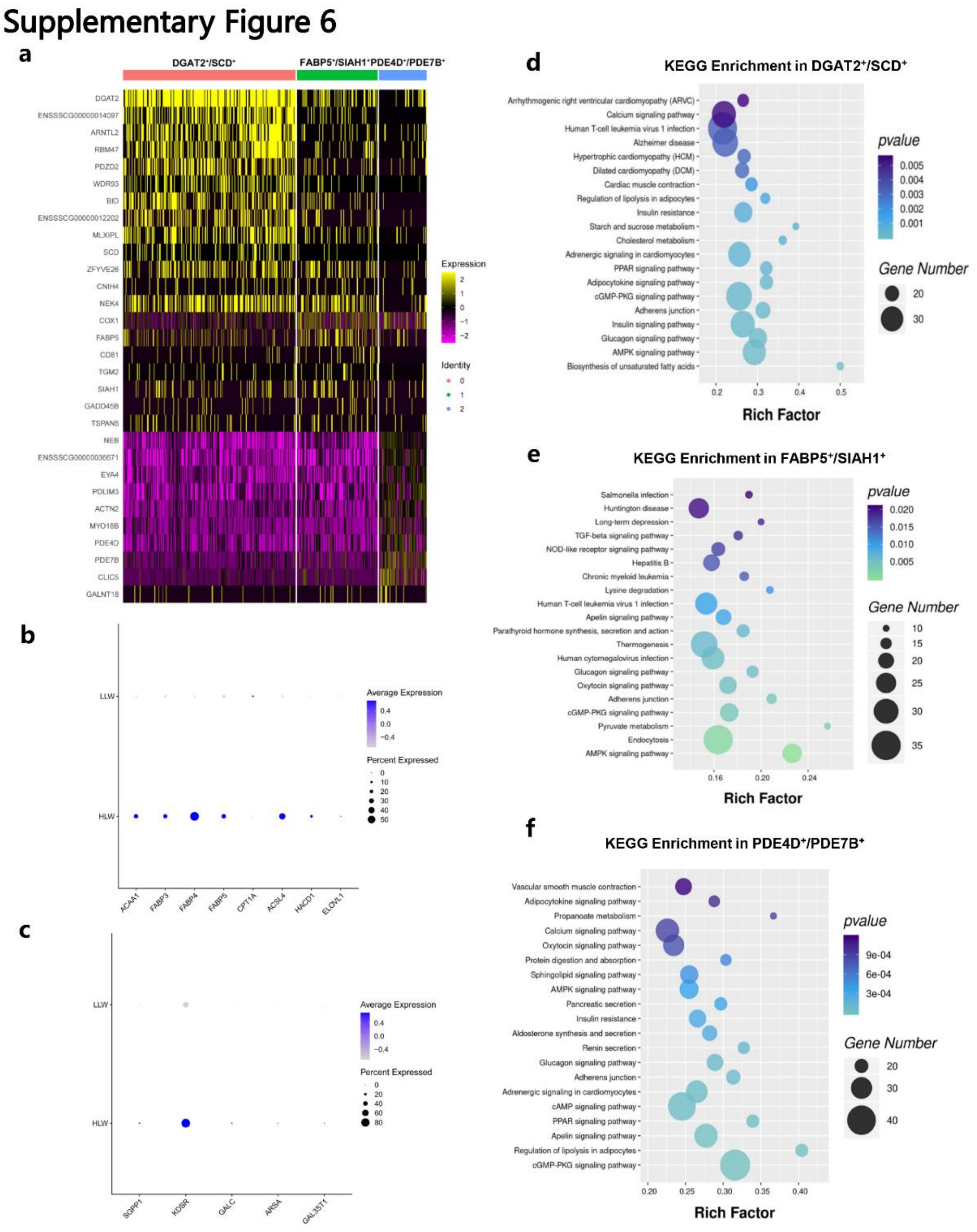

Supplementary Figure 6. Transcriptional heterogeneity in adipocytes nuclei. (a) Heatmap representing the top 10 most differently expressed genes between cell subclusters identified. **(b)** Dot plot showing the expression of fatty acid metabolism-related genes in the HLW and LLW groups. **(c)** Dot plot showing the expression of sphingolipid metabolism-related genes in the HLW and LLW groups. **(d)** KEGG enrichment analysis of genes in DGAT2$^+$/SCD$^+$ subcluster. **(e)** KEGG enrichment analysis of genes in FABP5$^+$/SIAH1$^+$ subcluster. **(f)** KEGG enrichment analysis of

genes in PDE4D⁺/PDE7B⁺ subcluster. Statistical analysis was performed using two-tailed Student's t-test.

Supplementary Figure 7

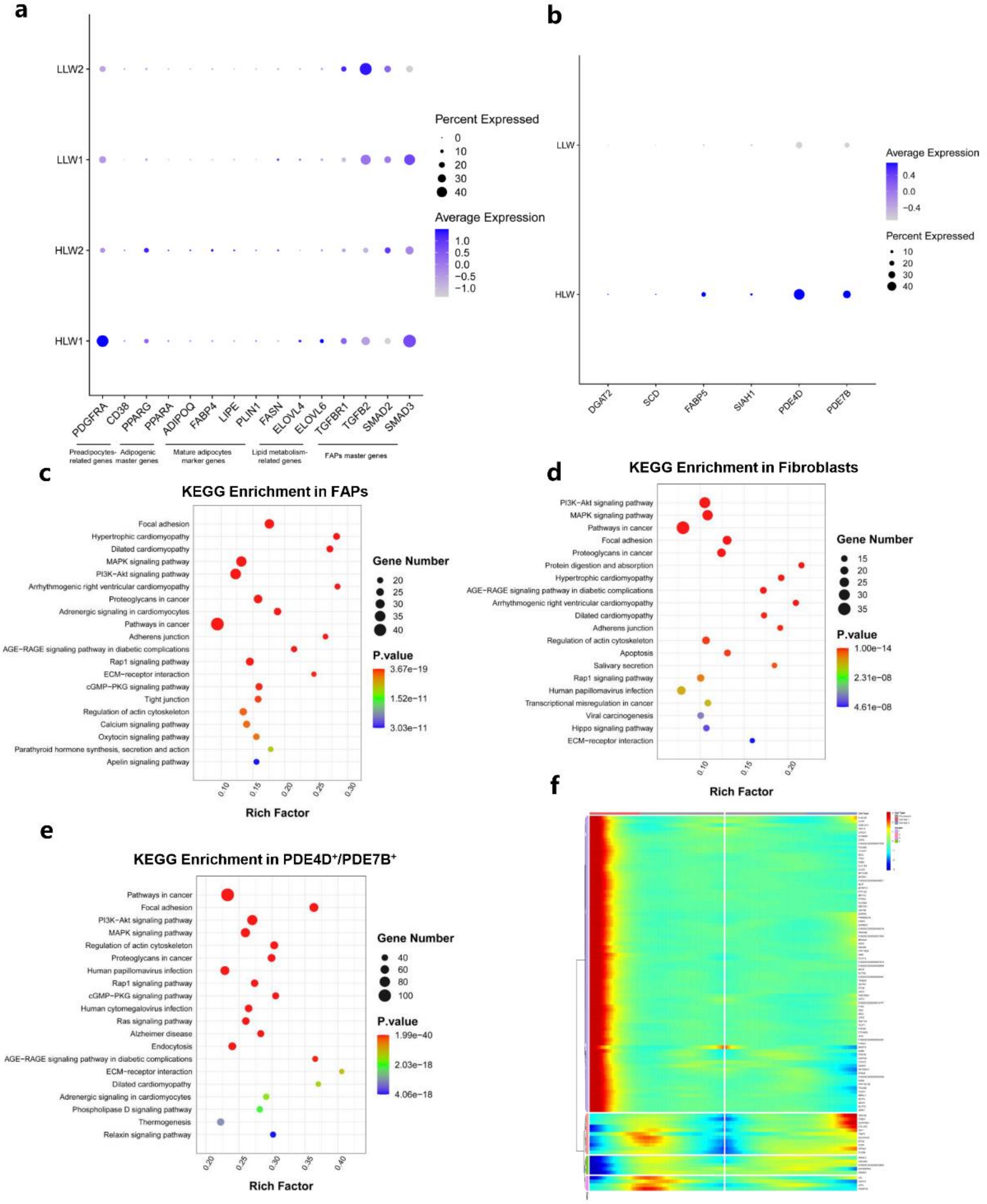

Supplementary Figure 7. Transcriptional heterogeneity in FAPs/fibroblasts nuclei. **(a)** Dot plot showing the expression of preadipocytes-related genes (*PDGFRA* and *CD38*), adipogenic master genes (*PPARG* and *PPARA*), mature adipocyte marker genes (*ADIPOQ*, *FABP4*, *LIPE*, and *PLIN1*), lipid metabolism-related genes (*FASN*, *ELOVL4*, and *ELOVL6*) and FAPs master genes (*TGFBR1*, *TGFB2*, *SMAD2*, and *SMAD3*) in

different group. **(b)** Dot plot showing the expression of marker genes for adipocytes subcluster of nuclei in different groups. **(c)** KEGG enrichment analysis of genes in FAPs subcluster. **(d)** KEGG enrichment analysis of genes in fibroblasts subcluster. **(e)** KEGG enrichment analysis of genes in PDE4D^{+}/PDE7B^{+} subcluster. **(f)** The pseudotemporal heatmap showing gene expression dynamics at Point 2. Statistical analysis was performed using two-tailed Student's t-test.

Supplementary Figure 8

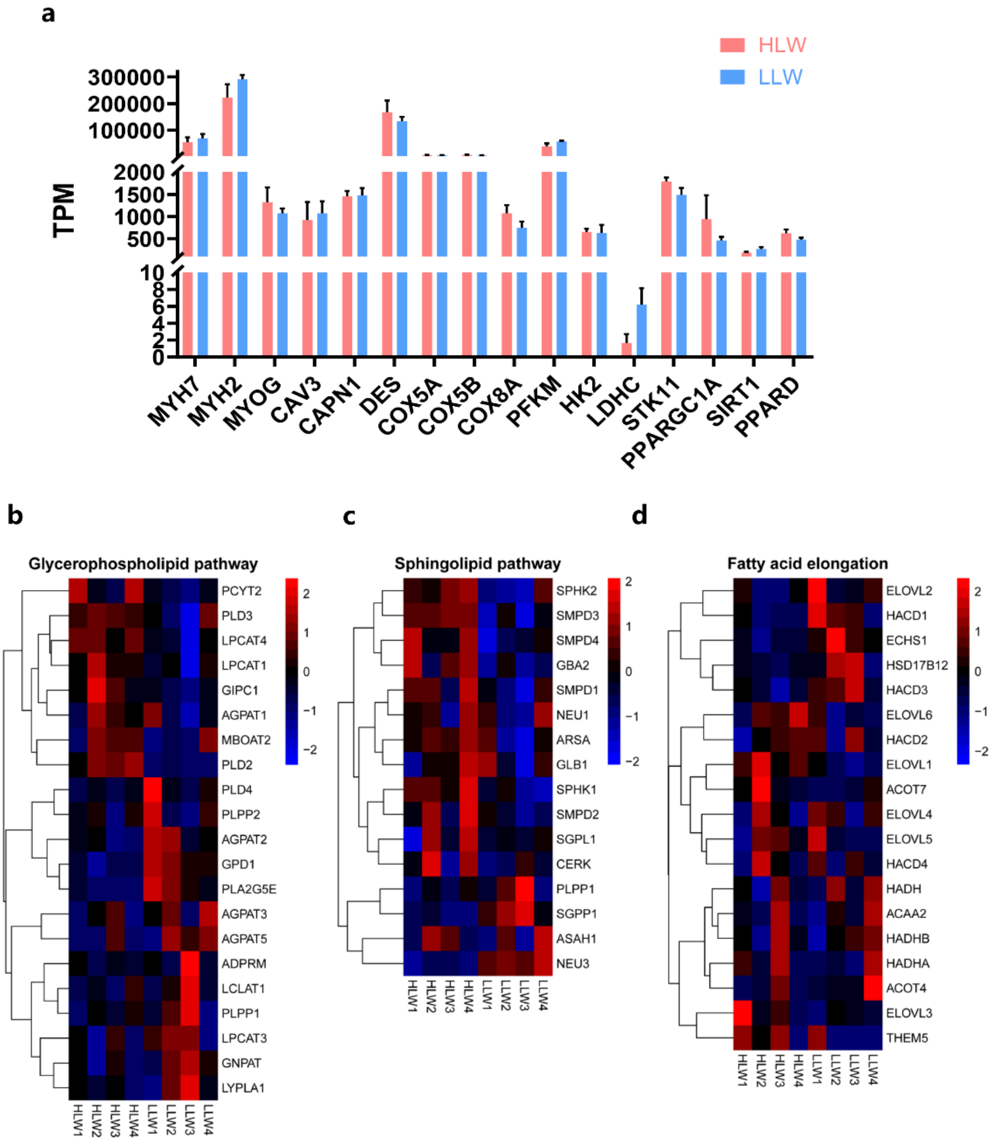

Supplementary Figure 8. Transcriptional dynamics between different groups. (a) TPM value of myogenesis-related genes (*MYH7*, *MYH2*, *MYOG*, *CAV3*, *CAPN1*, and *DES*), oxidation-related genes (*COX5A*, *COX5B*, and *COX8A*), glycolysis-related genes (*PFKM*, *HK2*, and *LDHC*), and myofiber type transformation-related genes (*STK11*, *PPARGC1A*, *SIRT1*, and *PPARD*) in different groups (n = 4). **(b-d)** The heatmap showing relative expression of the glycerophospholipid pathway **(b)**, biosynthesis of sphingolipid pathway **(c)**, and fatty acid elongation **(d)** related genes derived from the RNA-seq dataset. Error bars represent SEM, two-tailed Student’s t-test.
